# Retrosplenial cortical reorganization during late adolescence introduces instability of contextual memory circuits

**DOI:** 10.1371/journal.pbio.3003908

**Published:** 2026-07-17

**Authors:** Hui Zhang, Zorica Petrovic, Elizabeth M. Wood, Ana Cicvaric, Maayan Krispil-Alon, Vladimir Jovasevic, Kendra Parker, Thomas E. Bassett, Anna Carboncino, Anita L. Guedea, Pengfei Yi, Gal Richter-Levin, J. Tiago Gonçalves, Jelena Radulovic

**Affiliations:** 1 Dominick P. Purpura Department of Neuroscience, Albert Einstein College of Medicine, The Bronx, New York, United States of America; 2 Department of Pharmacology, Feinberg School of Medicine, Northwestern University, Chicago, Illinois, United States of America; 3 Department of Psychiatry and Behavioral Sciences, Feinberg School of Medicine, Northwestern University, Chicago, Illinois, United States of America; 4 Sagol Department of Neurobiology, University of Haifa, Haifa, Israel; 5 Department of Psychiatry and Behavioral Sciences, Psychiatry Research Institute Montefiore Einstein (PRIME), Albert Einstein College of Medicine, The Bronx, New York, United States of America; 6 Department of Biomedicine, Aarhus University, Aarhus, Denmark; Center for Brain Research, Medical University of Vienna, AUSTRIA

## Abstract

Hippocampal and cortical memory circuits, which enable the capacity to remember details from one’s past, are believed to reach maturity by early adolescence. Here, we demonstrate that the transition from early to late adolescence involves extensive reorganization of the retrosplenial (RSP) cortex along with significant memory expression deficits. Specifically, the densities of perineuronal nets (PNNs) and the expression of parvalbumin (PV) established in mice RSP during early adolescence (p30) significantly declined by late adolescence (p60–p75). In parallel, using context fear conditioning, we found that memories acquired during early adolescence were significantly impaired. These cellular and memory changes could be alleviated by PNN stabilization, indicating that they were secondary to PNN loss. Consistent with this, memory expression spontaneously recovered with PNN build-up in later life. Furthermore, late adolescence mice show a decrease in the level of key PNN constituents (especially aggrecan and neurocan) and the expression of transforming growth factor beta (Tgfβ) in RSP. RSP-targeted infusion of Tgfβ2 attenuated the decrease of PNN and neurocan, supporting a role of Tgfβ2 signaling in PNN build-up. These indicate that the decrease of PNNs during late adolescence was likely due to multiple molecular adaptations. Together, our findings show that RSP ECM undergoes dynamic reorganization until adulthood and beyond, with particularly strong fluctuations during late adolescence. In addition to affecting the expression of remote memories, in susceptible individuals, the observed dynamics could interact with genetic factors, increasing the risk of late adolescent psychopathologies.

## Abstract 2

Memory circuits are thought to mature by early adolescence, but how retrosplenial cortex development affects later memory expression remains unclear. This study shows that adolescence entails loss of perineuronal nets (PNN) and parvalbumin in retrosplenial cortex, which was paralleled by impaired memory expression that was recoverable through PNN stabilization and maturation.

## Introduction

Episodic and autobiographical memories, which enable individuals to consciously remember and re-experience past events [1,2], depend on the integrity and function of hippocampal and cortical networks [3,4]. In humans, this capacity emerges in early childhood and is believed to show a linear growth in its spatial, temporal, and item components [5]. Brain development in early childhood has been studied extensively, not least because of the well-established “loss” of early childhood memories, a phenomenon known as infantile amnesia. However, there is mounting evidence indicating profound adolescent changes of brain structure and function, especially within cortical areas [6]. Adolescent cortical maturation involves changes of gray matter [7–9], decrease of low-frequency EEG power [10,11], decrease in cortical neuron complexity [12], and changes of structural connectivity [13]. These changes are likely to affect the cognitive processes unique to the adolescent period [14,15], especially as they pertain to the field of stress-related memories. The field has been sharply divided for decades between researchers who hold the position that adolescent memories are remembered the best [16] and those who hold the position that such memories are not well remembered [17]. Understanding the molecular mechanisms underlying these changes could provide important insights not only into the dynamics of adolescent memories, but also into vulnerabilities to psychopathologies emerging during this period.

Rodent studies suggest that at the neurobiological level, episodic-like memories emerge from specific patterns of neuronal activity distributed across hippocampal and cortical networks, including the entorhinal, prefrontal, and retrosplenial (RSP) cortices [18–20]. The ability to reconstruct these patterns through neuronal reactivation is achieved, among other mechanisms, through condensation of the extracellular matrix (ECM) into mesh-like structures known as perineuronal nets (PNNs), mainly formed around parvalbumin (PV) interneurons [21–23]. PNNs are composed of condensed chondroitin sulphate proteoglycans (also known as lecticans) [24], whose levels are controlled by pleiotropic actions of growth factors, especially Tgfβ1 and Tgfβ2 [25], as well as various enzymes and immune mediators [26]. PNNs are believed to stabilize neuronal circuits by several mechanisms, such as limiting access to interfering synaptic inputs and stabilizing local inhibition [21,27]. There is also evidence that PNNs play a key role in PV interneuron viability, by protecting them from reactive metabolites generated during their high spiking activity [28].

The build-up of PNNs is viewed as critical for the maturation of memory circuits, underlying their ability to retain information over long periods of time [23,29]. Based on hippocampal studies, it is widely believed that PNNs in mice reach adult levels between postnatal days 24–28 (p24–p28), corresponding to early adolescence in humans [30–32]. The cortical PNN dynamics at later developmental periods, especially with respect to memory functions, are less well understood. Here, we studied the dynamics of PNNs and interneurons from early adolescence to middle adulthood (aligned with human developmental age based on current considerations, as depicted in S1A Fig). We focused on RSP because of its direct interaction with the dorsal hippocampus (DH) and dorsal subiculum (SUB) [33–35], its unique, time-independent contribution to memory expression [36–40], and its key role in driving episodic [41,42] and default mode [43,44] networks. We demonstrated that the PNN density and PV expression established in early adolescence showed RSP-specific downregulation during late adolescence. These changes were followed by significant expression deficits of context memories acquired during early adolescence. Stabilization of the extracellular matrix and PNN levels, and partly an increase of PNN in later life, alleviated the reduction of PV interneurons and memory expression, suggesting that the latter changes were secondary to PNN degradation. PNN degradation, on the other hand, was associated with RSP-intrinsic reductions of lectican levels and TGFβ expression. Together, these findings identify late adolescence as a critical period of ECM dynamics introducing instability and, possibly, vulnerability in a cortical area driving key memory and affective networks.

## Results

### Changes of PNN densities in the RSP-DH circuit at the transition between early and late adolescence

**Fig 1:**
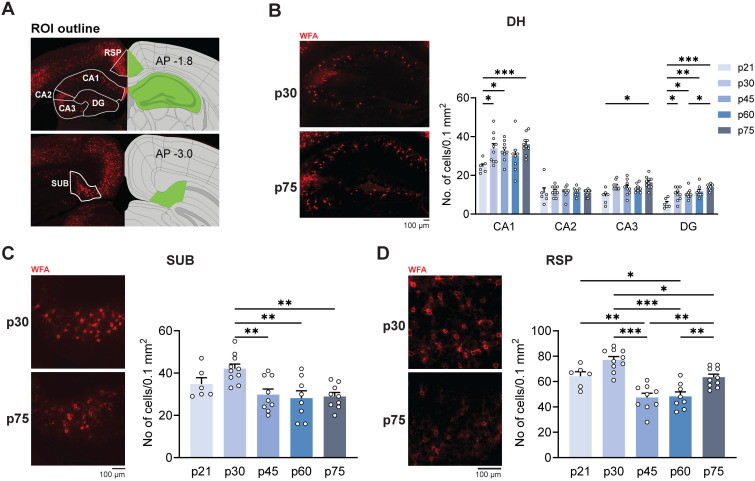
PNN build-up in RSP, DH, and SUB throughout adolescence. **(A)** The outline of ROI for RSP, DH, and SUB. Representative images of region-specific WFA staining (left) and quantification for the density of PNN^+^ cells (right) show (B–D): (**B)** Stable or increasing density of PNNs in DH (p21: *n* = 6, p30 and p75: *n* = 10, p45: *n* = 9, p60: *n* = 8; two-way RM ANOVA: Region: *F*_(2.146, 81.62)_ = 246.2 *p* < 0.001, Age: *F*_(4, 38)_ = 9.717 *p* < 0.001, Region × Age: *F*_(12,114)_ = 1.719 *p* = 0.071; Tukey’s post-hoc test: CA1: p21 vs. p30: *p* = 0.029, p21 vs. p45: *p* = 0.017, p21 vs. p75: *p* < 0.001, CA3: p21 vs. p75: *p* = 0.016, DG: p21 vs. p30: *p* = 0.032, p21 vs. p45: *p* = 0.019, p21 vs. p60: *p* = 0.010, p21 vs. p75: *p* < 0.001, p45 vs. p75: *p* = 0.020) in CA1, CA2, CA3, and DG. **(C)** Significant decrease in the density of PNNs in SUB (p21: *n* = 6, p30: *n* = 10, p45 and p75: *n* = 9, p60: *n* = 8; one-way ANOVA: *F* = 5.556, *p* = 0.001; Tukey’s post-hoc test: p30 vs. p45: *p* = 0.009, p30 vs. p60: *p* = 0.004, p30 vs. p75: *p* = 0.005). **(D)** Significant decrease in the density of PNNs in RSP during late adolescence (p21: *n* = 6, p30 and p75: *n* = 10, p45: *n* = 8, p60: *n* = 9; one-way ANOVA: *F* = 17.28, *p* < 0.001; Tukey’s post-hoc test: p21 vs. p45: *p* = 0.009, p21 vs. p60: *p* = 0.019, p30 vs. p45: *p* < 0.001, p30 vs. p60: *p* < 0.001, p30 vs. p75: *p* = 0.014, p45 vs. p75: *p* = 0.004, p60 vs. p75: *p* = 0.009). Data represent mean ± s.e.m., ***p < 0.001; **p < 0.01; *p < 0.05. The data underlying this Figure can be found in S1 Data.

We visualized PNNs with *wisteria floribunda agglutinin* (WFA) labeling, and quantified them in the DH, dorsal SUB, and RSP (Fig 1A) from pre-adolescence to late adolescence (p21–p75). The density (reported as number of cells/0.1 mm^2^) of PNNs in DH (Fig 1B) remained stable in CA2 and even increased with age in CA1, CA3, and dentate gyrus (DG). On the other hand, in SUB and RSP of both males and females (Figs 1C, 1D, and S1), PNN densities were high at p21 and p30, then significantly decreased between p45 and p75 (age effect: SUB: *p* = 0.001, RSP: *p* < 0.001).

These findings suggested that PNNs in RSP were established by early adolescence (p21). But as the DH progressively matured, SUB and RSP demonstrated significant PNN reductions, suggesting possible circuit instability in RSP.

### Late adolescent changes of RSP PV expressions

**Fig 2:**
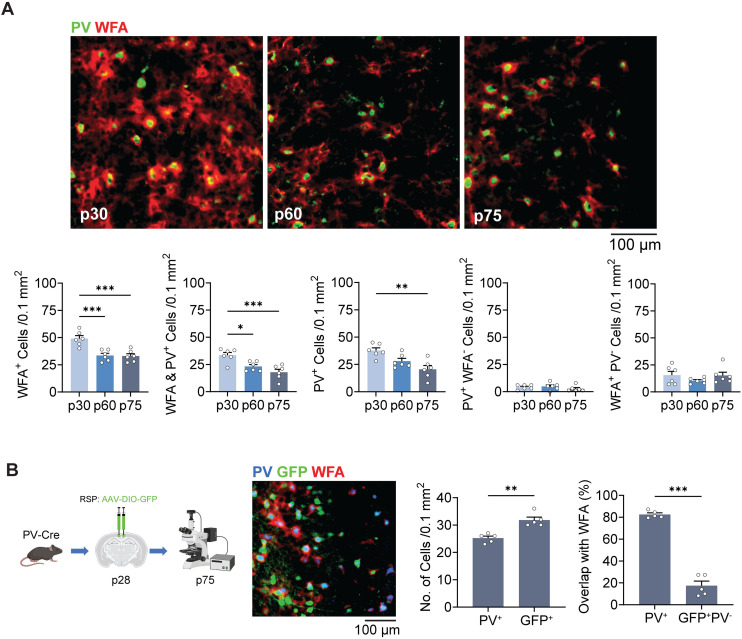
Change of RSP PV expressions in late adolescence. **(A)** Density and colocalization of WFA and PV-positive interneurons from adolescence to mid-adulthood revealing a significant late adolescent decrease (WFA^+^ cells: *n* = 6; one-way ANOVA: *F* = 14.85 *p* < 0.001, Tukey’s post-hoc test: p30 vs. p60: *p* < 0.001, p30 vs. p75: *p* < 0.001. WFA and PV^+^ cells: *n* = 6; one-way ANOVA: *F* = 11.13 *p* = 0.001; Tukey’s *p*ost-hoc test: p30 vs. p60: *p* = 0.020, *p*30 vs. p75: *p* < 0.001. PV^+^ cells: *n* = 6; one-way ANOVA: *F* = 8.854 *p* = 0.003; Tukey’s *p*ost-hoc test: p30 vs. p75: *p* = 0.002. PV^+^ WFA^-^ cells: *n* = 6; one-way ANOVA: *F* = 0.9638 *p* = 0.404). **(B)** Cre-de*p*endent AAV labeling of PV-interneurons at p28 shows fewer GFP-labeled cells show PV expression by p75 (*n* = 5, unpaired two-tailed *t* test, *t*_8_ = 4.947, *p* = 0.001) and lower PNN coverage in cells *t*hat lost PV ex*p*ression (*n* = 5, unpaired *t*wo-tailed *t* test, *t*_8_ = 14.76, *p* < 0.001). Ex*p*erimen*t*al diagrams were created in BioRender. Zhang, H. (2026) https://BioRender.com/upnrtln. Data represent mean ± s.e.m., ***p < 0.001; **p < 0.01; *p < 0.05. The data underlying this Figure can be found in S1 Data.

We next investigated whether the decrease of PNNs might be related to changes of RSP PV interneurons. Quantification revealed that the density of PV^+^ interneurons closely paralleled the changes of PNNs, showing a significant decrease at p60 and further reduction by p75 (ANOVA main effect: WFA^+^: *p* < 0.001, WFA and PV^+^: *p* = 0.001, PV^+^: *p* = 0.003, Fig 2A).

Developmental expansion of the entire mouse cortex has been reported during adolescence [45], which could contribute to the observed decrease of PNN and PV densities. Therefore, we estimated the volume change of targeted RSP region using two independent reference datasets, the Allen Developing Mouse Brain Atlas (ADMBA) [46] and the EBRAINS Developmental mouse brain atlas (DeMBA) [47]. Interestingly, both datasets showed a decrease, rather than an expansion, in RSP volume at p56 compared with p28 (ADMBA: 6.75% decrease; DeMBA: 7.43% decrease). These findings do not support the possibility that expansion of the targeted RSP region accounts for the observed decrease in PNN and PV densities.

We further asked if the reduced PV signal is due to the developmental change of PV expression within PV interneurons or the loss of these neurons. We injected AAV vector carrying Cre-dependent GFP into the RSP of PV-Cre mice at p28, labeling PV interneurons throughout this developmental period. At p75, we observed a lower density of immunolabeled PV^+^ cells compared to GFP^+^ cells in the RSP of these animals (*p* = 0.001, Fig 2B). This indicates that a subset of the cells previously expressed PV showed reduced PV expression by p75, contributing to the observed reduction in PV immunoreactivity. In addition, we observed low PNN coverage among cells that previous express PV but no longer showed detectable PV immunoreactivity (GFP^+^PV^−^) compared with cells that maintained current PV expression (PV^+^, *p* < 0.001, Fig 2B). These results suggest that reduced PNN expressions may be associated with the developmental changes in PV expressions. The reduction of the PV expression in late adolescent RSP was specific for this cell population and did not involve similar changes of microglia, as found in the prefrontal cortex around p60 [48], whereas astrocytes and oligodendrocytes did not show a similar decline and even slightly increased between early and late adolescence (*p* < 0.001, S2 Fig).

### Effects of late adolescent RSP reorganization on recent and remote memory expression

**Fig 3:**
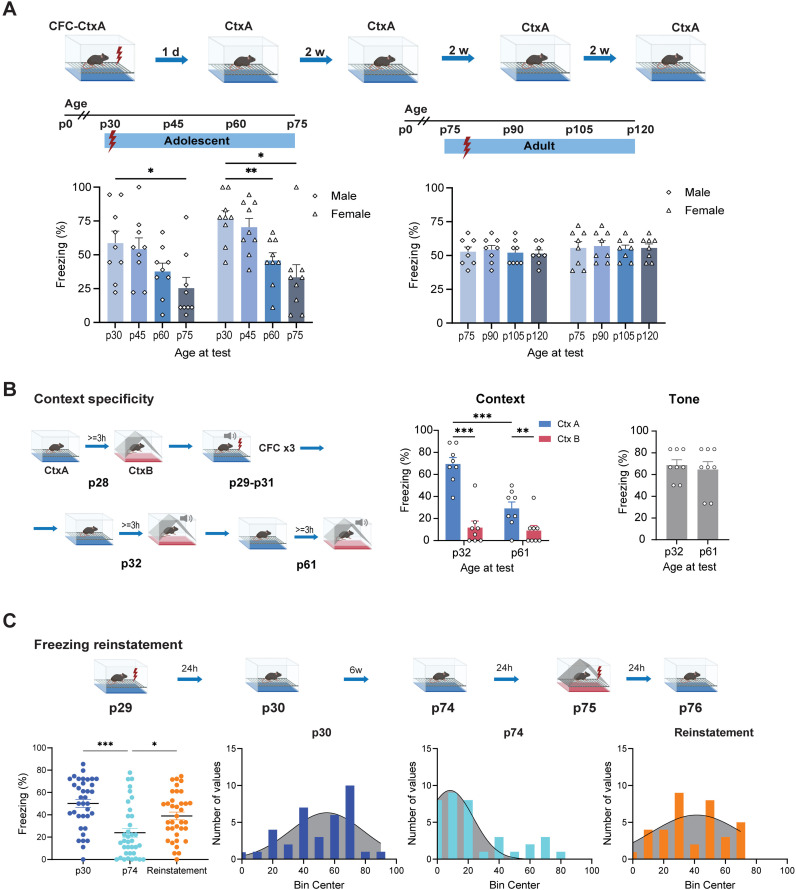
Expression of aversive context memories throughout adolescence. **(A)** Both male and female mice that underwent CFC on p29 (adolescent, bottom left) showed reduced freezing 6 weeks after CFC (*n* = 9/group for each sex, Two-way RM ANOVA, Sex: *F*_(1,16)_ = 2.715 *p* = 0.119, Age of test: *F*_(2.114,33.83)_ = 17.62 *p* < 0.001 Sex × Age of test: *F*_(2.114,33.83)_ = 0.3761 *p* = 0.701), while mice conditioned on p75 (adult, bottom right) showed stable freezing throughout 6 weeks (*n* = 8 for each sex, Two-way RM ANOVA, Sex: *F*_(1, 14)_ = 0.6921 *p* = 0.419, Age of test: *F*_(2.004, 28.61)_ = 0.3052 *p* = 0.744 Sex × Age of test: *F*_(2.004, 28.61)_ = 0.03808 *p* = 0.965). **(B)** The context discrimination task with tests performed p32 and p61 (left) showed that the fear memory was context specific. The impairment was not due to extinction because it was also robust in the absence of repeated testing (top right; *n* = 8; two-way RM ANOVA: Age of test: *F*_(1,7)_ = 35.22 *p* < 0.001, Context: *F*_(1, 7)_ = 48.14 *p* < 0.001 Age of test × Context: *F*_(1,7)_ = 42.88 *p* < 0.001; Šídák’s post-hoc test: p32 CtxA vs. p61 CtxA: *p* < 0.001, p32 CtxA vs. CtxB: *p* < 0.001, p61 CtxA vs. CtxB: *p* = 0.01). Freezing levels to auditory cue did not change with time (Bottom right; paired two-tailed *t* test: *t*_7_ = 0.6831, *p* = 0.516). **(C)** Summary of two independent experimen*t*s (a total of *n* = 36) demons*t*rating robust loss and reinstatement of adolescent fear memory in ~75% of the tested mice, as illustrated by individual freezing values (one way ANOVA *F*_(2, 105)_ = 12.63, *p* < 0.001, bottom left) and frequency distribution analyses of freezing behavior on p30, p74, and p76 (bottom middle and right). Experimental diagrams were created in BioRender. Zhang, H. (2026) https://BioRender.com/8fr6chl. Data represent mean ± s.e.m., ***p < 0.001; **p < 0.01; *p < 0.05. The data underlying this Figure can be found in S1 Data.

To better understand the potential impact of the observed RSP changes on memory functions, we measured the formation and expression of stress-induced context memories acquired during early or late adolescence. We first trained the mice in one-trial CFC (3 min CtxA exposure, 2 s footshock, 0.7 mA, constant current) on p29 or p75 and tested them every two weeks thereafter for memory-induced freezing behavior in response to context re-exposure. Mice of both sexes trained on p29 showed impaired CFC memory by p75, as revealed by a significant decrease of their freezing levels (age at test effect: *p* < 0.001), whereas mice trained at p75 retained their memories within a similar 6-week time frame by p120 (age at test effect: *p* = 0.744, Fig 3A). This effect was replicated in a follow-up experiment whose objectives were to determine: (i) whether the impairment might have been due to enhanced extinction induced by repeated testing; (ii) whether the adolescent memory lacked specificity that might have affected its persistence; and (iii) whether hippocampus-independent associative memories, such as delay tone-dependent fear conditioning, undergo similar impairments. We exposed the mice on p28 to a modified fear conditioning paradigm allowing for the determination of context specificity/discrimination [49,50] and for assessment of both CFC and delay tone-dependent fear conditioning (Fig 3B). The paradigm consisted of a 3-min pre-exposure to CtxA and CtxB. Mice were then fear conditioned by exposing themto CtxA (3 min), tone (10 kHz, 30 s), and footshock (0.7 mA constant current, 2 s), once a day over 3 days. Memory tests performed one day (p32) or one month later (p61) replicated the significant impairment of early adolescent context-shock memory (Fig 3B, middle, p32 versus p61 in CtxA: *p* < 0.001) without affecting the tone-shock memory (Fig 3B, right, *p* = 0.516), which does not depend on the DH-RSP circuit. At all ages between p25 and p65, mice showed an intact ability to differentiate between CtxA and CtxB where shock was never delivered (p32: *p* < 0.001; p61: *p* = 0.036, also see S3 Fig). The inability to express a previously acquired context memory was unlikely due to adolescent changes of fear or anxiety expression, given that mice trained during late adolescence were able to form long-lasting, context-specific memories (S4 Fig). We performed additional experiments to examine whether the observed PNN dynamics were caused by RSP-intrinsic mechanisms or by circuit adaptations caused by the maturation of hippocampal-RSP projections. Using reporter mice expressing green fluorescent protein (GFP) driven by the neurotensin (*NTS*) gene to label DH/SUB to RSP projections, we found that indeed, DH maturation involved changes in the connectivity between the SUB and RSP (especially disappearance of a SUB-RSP layer 5 projection, S5A–S5C Fig). However, despite these circuit adaptations, inactivation of DH-RSP projections showed no interference with the expression deficits of early adolescent memories (S5D Fig).

Two subsequent experiments were devised to determine whether the observed memory impairments reflected memory degradation, or limited memory access. In the first study, we investigated whether early adolescent context memories can be reinstated as a function of re-experiencing the footshock in a new context (CtxB). The second study was devised to provide a replicate of the reinstatement effect, with an additional, no-shock control. Data from both studies were presented together in Fig 3C, whereas findings for each experiment separately are shown in S6 Fig. Overall, the findings replicated the adolescent memory expression deficit (p30 versus p74: *p* < 0.001), but after subsequent CFC in CtxB, freezing to CtxA significantly increased (p74 versus p76 reinstatement test: *p* = 0.014), indicating reinstatement of CtxA memory. This effect was not found in no shock controls, ruling out CtxB-induced generalization (S6 Fig).

Together, these findings demonstrated that although stress-induced context memories acquired during early adolescence are initially robust and specific, they showed delayed expression deficits (relative to memories acquired during adulthood), coinciding with a decrease of RSP PNN and PV interneuron densities. The impairment was sex-independent and not due to memory loss, inability to express fear, or general impairments in retrieving negative associative memories.

### Effect of RSP PNN stabilization on cellular and memory impairments during adolescence

**Fig 4:**
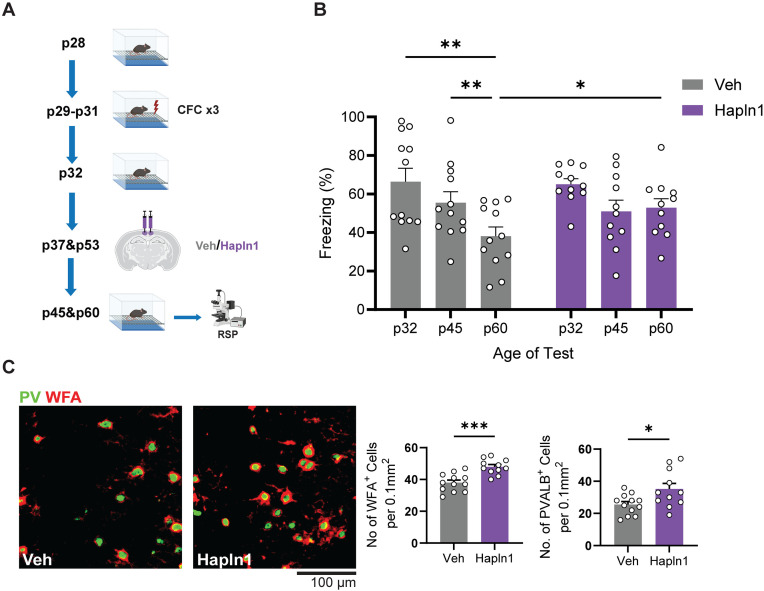
Effect of PNN stabilization during adolescence on PNN build-up, PV expression, and memory. **(A)** Experimental paradigm for HAPLN1 injection. **(B)** Injection of HAPLN1 prevented the time-dependent reduction of freezing (Veh: *n* = 12, HAPLN1: *n* = 11, two-way RM ANOVA: Age at test: *F*_(1.933,41.86)_ = 14.70 *p* < 0.001, HAPLN1: *F*_(1,21)_ = 0.2303 *p* = 0.636, Age at test × HAPLN1: *F*_(1.993,41.86)_ = 3.761 *p* = 0.032, Šídák’s post-hoc test: Veh: p32 vs. p60: *p* = 0.002, p45 vs. p60: *p* = 0.007; p60: Veh vs. HAPLN1: *p* = 0.038). **(C)** Representative images (top) and quantifications demonstrated significantly increased WFA^+^ cell densities (bottom left, Veh: *n* = 12, Hapln1: *n* = 11, unpaired two-tailed *t t*est: *t*_21_ = 4.577 *p* < 0.001) and PV^+^ cells (bottom right, Veh: *n* = 12, Hapln1: *n* = 11, unpaired two-tailed *t* tes*t*: *t*_21_ = 2.490 *p* = 0.0212) in the RSP of HAPLN1-injec*t*ed mice. Experimental diagrams were created in BioRender. Zhang, H. (2026) https://BioRender.com/th31zfo. Data represent mean ± s.e.m., *** *p* < 0.001; ** *p* < 0.01; * *p* < 0.05. The data underlying this Figure can be found in S1 Data.

To determine whether the late reduction of remote context memory expression and PV expressions were directly linked to PNN degradation, we next investigated whether their decrease can be prevented by stabilizing PNNs using RSP-targeted injections of hyaluronan and proteoglycan link protein 1 (HAPLN1, Fig 4A). This approach was selected based on the documented efficacy of HAPLN1 to stabilize the ECM in various in vivo models [51,52]. Injections of HAPLN1 alleviated the impairments of adolescent memory expression (Age of test × HAPLN1 interaction: *p* = 0.032, p60: Veh versus Hapln1: *p* = 0.038), the degradation of PNNs (*p* < 0.001), and the reduction of cells showing PV expression (*p* = 0.021) relative to vehicle (Veh)-injected control mice (Fig 4B and 4C). These findings indicated that PNN degradation directly contributed to the loss of PV expressions, identifying PV interneurons as a vulnerable cell population with respect to changes of cortical ECM dynamics, and that remote memory expression deficits were secondary to the PNN/PV decline.

We also explored whether early adolescent context memories might spontaneously recover as mice age. To this end, we extended the period for memory assessment and observed a significant spontaneous recovery by middle adulthood (p120–p150). Memory recovery was accompanied by substantial generalization, as revealed by similar freezing in previously shock-associated CtxA and novel context CtxB (p30 versus p90: *p* < 0.001, p90 versus p150: *p* = 0.002, p90 versus p180: *p* = 0.012, p180/CtxA versus p181/CtxB: *p* = 0.997, S7A Fig). To determine whether further PNN/PV changes could account for these effects, we quantified the densities of RSP PNNs and PV^+^ cells on p30 and p150. Unexpectedly, the densities of PNNs and PV^+^ cells on p150 not only recovered but exceeded the levels found in early adolescence. However, there was a substantial increase of the proportion of WFA-negative PV interneurons at p150 relative to p30 (*p* < 0.001, S7B Fig). This was not due to the formation of PNNs around other main interneuron classes (S7C Fig, p30 versus p150: PV: *p* < 0.001 SST: *p* = 0.999, VIP: *p* = 0.690, NPY: *p* > 0.999, CR: *p* > 0.999). This supported our findings linking PNNs to memory expression, but also suggested that the memory fidelity might be additionally dependent on the PNN/PV interneuron overlap.

### RSP PNN-associated lectican levels from early to late adolescence

**Fig 5:**
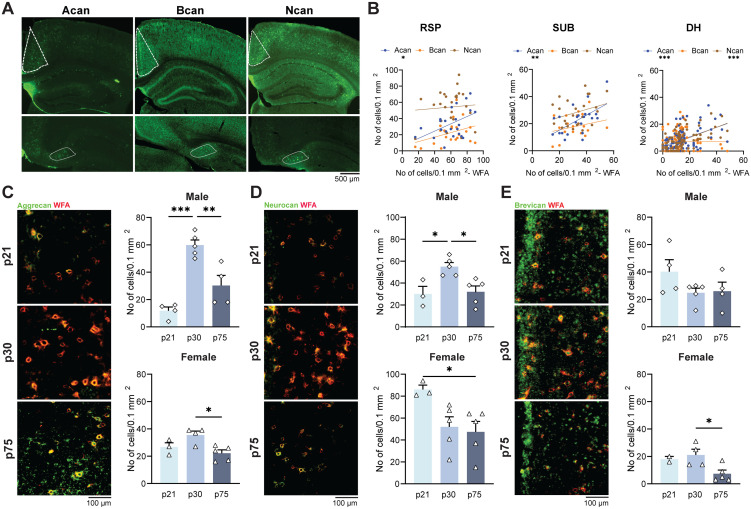
Fluctuations of lectican levels throughout adolescence. **(A)** Regions of interest (ROI) for RSP immunolabeling analyses of aggrecan (Acan, left), brevican (Bcan, middle), and neurocan (Ncan, right). **(B)** Linear regression analysis of the density of cells with individual PNN lecticans and the density of WFA^+^ cells in RSP (Acan: *R*^2^ = 0.229, *F*_(1,21)_ = 6.226, *p* = 0.021), SUB (Acan: *R*^2^ = 0.337, *F*_(1,20)_ = 10.16, *p* = 0.005), and DH (Acan: *R*^2^ = 0.251, *F*_(1,94)_ = 31.56, *p* < 0.001, Ncan: *R*^2^ = 0.349, *F*_(1,102)_ = 54.70, *p* < 0.001). **(C)** Left: representative image of aggrecan (green) and WFA (red) labeling in RSP. Right: quantification of the density of WFA^+^ aggrecan+ cells in RSP showing significant decrease in males (top; p30: *n* = 5, others: *n* = 4; one-way ANOVA: *F* = 25.75, *p* < 0.001; Tukey’s post-hoc test: p21 vs. p30: *p* < 0.001, p30 vs. p75: *p* = 0.004) and females (bottom; p21: *n* = 3, p30: *n* = 4, p75: *n* = 5; one-way ANOVA: *F* = 6.475, *p* = 0.018; Tukey’s post-hoc test: p30 vs. p75: *p* = 0.015). **(D)** Left: representative image of neurocan (green) and WFA (red) labeling in RSP. Right: quantification for the density of WFA^+^ neurocan+ cells in RSP with reduced neurocan expression in males (top; p21: *n* = 3, others: *n* = 5; one-way ANOVA: *F* = 7.686, *p* = 0.010; Tukey’s post-hoc test: p21 vs. p30: *p* = 0.023, p30 vs. p75: *p* = 0.017) and females (bottom; p21: *n* = 3, others: *n* = 5; one-way ANOVA: *F* = 4.319, *p* = 0.045; Tukey’s post-hoc test: p21 vs. p75: *p* = 0.046). **(E)** Left: representative image of brevican (green) and WFA (red) labeling in RSP. Right: quantification of the density of WFA^+^ brevican+ cells in RSP with levels in males (top; p30: *n* = 5, others: *n* = 4; one-way ANOVA: *F* = 1.855, *p* = 0.207) and significant reduction in females (bottom; p21: *n* = 2, p30: *n* = 4, p75: *n* = 5; one-way ANOVA: *F* = 4.928, *p* = 0.040; Tukey’s post-hoc test: p30 vs. p75: *p* = 0.040). Data represent mean ± s.e.m., ***p < 0.001; **p < 0.01; *p < 0.05. The data underlying this Figure can be found in S1 Data.

We next investigated possible causes of PNN loss by examining the levels of key PNN constituents-the lecticans aggrecan (Acan), neurocan (Ncan), brevican (Bcan), and phosphacan (Pcan). The detection specificity of these proteoglycans (Fig 5A) was validated by the significant correlation between WFA and Acan in RSP (*R*^2^ = 0.229, *p* = 0.002) and SUB (*R*^2^ = 0.337, *p* = 0.005) and with Acan (*R*^2^ = 0.251, *p* < 0.001) and Ncan (*R*^2^ = 0.349, *p* < 0.001) in DH (Fig 5B). Age-dependent analysis revealed a profound and persistent decrease of Acan (Fig 5C: p30 versus p75: *p* = 0.001) and Ncan (Fig 5D, male: p30 versus p75: *p* = 0.017, female: p21 versus p75 *p* = 0.046) in PNNs of both p75 males and females relative to pre-adolescent (p21) or early adolescent (p30) mice. Bcan levels were also reduced in females (Fig 5E, p30 versus p75: *p* = 0.040). In addition, we observed a decrease of Pcan across RSP layers (S8 Fig). Although lecticans in the DH also showed some age-related changes, they mainly involved increased incorporation of Acan and Ncan in PNNs (S9A Fig, age effect: Acan male: *p* = 0.011, Acan female: *p* = 0.039, Ncan male: *p* < 0.001), whereas SUB showed similar decreases as RSP (S9B Fig, age effect: Acan male: *p* = 0.022, Acan female: *p* < 0.001, Ncan male: *p* = 0.036). Pcan (age effect: DH: male: *p* < 0.001, female *p* = 0.001, SUB: male: *p* = 0.003, female *p* < 0.001) and Bcan (in females only, DH: *p* < 0.001, SUB: *p* = 0.006) showed age-related decrease in all extracellular matrix compartments.

The late adolescent decrease of lectican levels was consistent the decrease of PNN densities during late adolescence and could contribute, at least in part, to their degradation. Interestingly, although PNN levels, PV expressions, and memory deficits were similar in males and females, individual lectican levels (e.g., Bcan in females) can be influenced by sex-specific factors.

### Late adolescent changes of Tgfβ and Tgfβ-mediated effects on PNN and lectican levels

**Fig 6:**
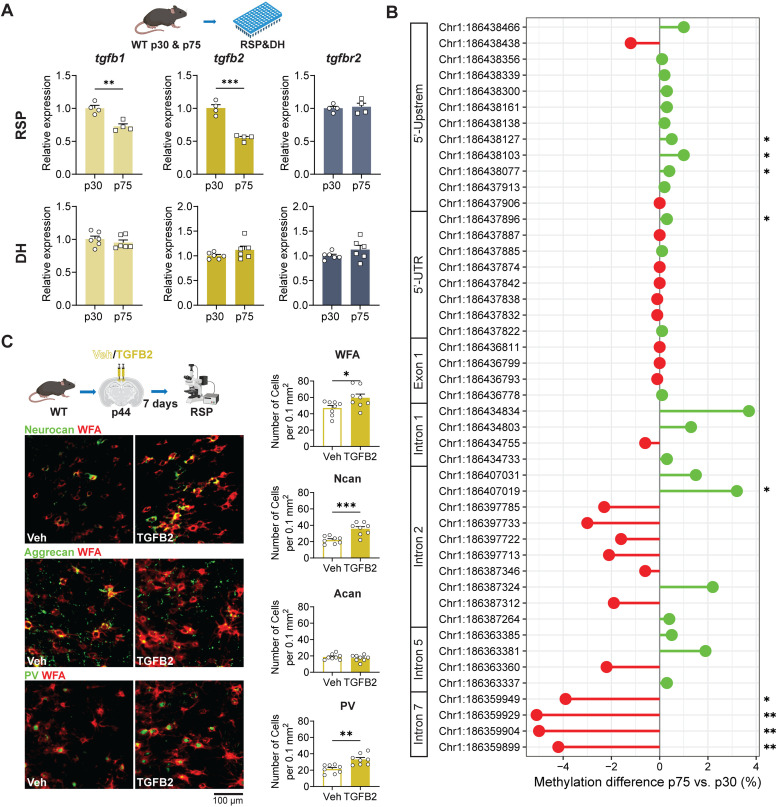
Developmental changes of Tgfβ expression and its effects on PNN and lectican levels. **(A)** The expression of *Tgfb1* and *Tgfb2* mRNA decreases in the RSP but not hippocampus between p30 and p75 (unpaired *t* test: RSP *Tgfb1*: *n* = 4, *t*_6_ = 4.723 *p* = 0.003; RSP *Tgfb2*: *n* = 4, t_6_ = 7.966, *p* < 0.001). **(B)** The methylation change between p75 and p30 on selected CpG islands of *Tgfb2* gene, significant marks represent significant differences between p30 and p75 percentage of methylation on corresponding CpG islands (*n* = 4, Chr1:186438127: *t*_6_ = 2.756, *p* = 0.033, Chr1:186438103: *t*_6_ = 3.231, *p* = 0.018, Chr1:186438077: *t*_6_ = 2.466, *p* = 0.049, Chr1:186437896: *t*_6_ = 2.449, *p* = 0.050, Chr1:186407019: *t*_6_ = 3.209, *p* = 0.018, Chr1:186359949: *t*_6_ = 2.992, *p* = 0.024, Chr1:186359929: *t*_6_ = 5.222, *p* = 0.002, Chr1:186359904: *t*_6_ = 4.877, *p* = 0.003, Chr1:186359899: *t*_6_ = 4.129, *p* = 0.006). **(C)** In*t*ra-RSP Tgfβ2 injection attenuates the loss of PNNs (*n* = 8; unpaired *t t*est: *t*_14_ = 2.278, *p* = 0.039), Ncan (*n* = 8; unpaired *t t*est: *t*_14_ = 4.325, *p* < 0.001), and PV (*n* = 8; unpaired *t* tes*t*: *t*_14_ = 3.937, *p* = 0.002) expressions one week later. Experimen*t*al diagrams were created in BioRender. Zhang, H. (2026) https://BioRender.com/tgo8isf. Data represent mean ± s.e.m., ***p < 0.001; **p < 0.01; *p < 0.05. The data underlying this Figure can be found in S1 Data.

Previous studies have shown that lectican gene expression is controlled through extracellular levels of active Tgfβ1 and Tgfβ2 [25,53]. We therefore examined whether this pathway could be linked to some of the observed PNN and lectican changes. A qPCR analysis revealed a significant reduction of Tgfβ, and especially *Tgfb2* mRNA expression in RSP of p75 mice compared to p30, whereas no significant change was observed in DH in the same period (RSP p30 versus p75: *Tgfb1*: *p* = 0.003, *Tgfb2*: *p* < 0.001, Fig 6A). These changes were associated with epigenetic changes of the *Tgfb2* gene, as revealed by multiple, bidirectional changes of *Tgfb2* gene body methylation (Fig 6B). To determine whether restoring Tgfβ2 levels could reverse some of the late adolescent changes of the extracellular matrix, we performed one RSP injection of Tgfβ2 at p44 (at the time of first detection of PNN decrease, Fig 1D), and quantified the levels of PNNs, the most significantly affected lecticans (Ncan and Acan), and PV one week later. A single RSP infusion of Tgfβ2 on p44 significantly increased the PNN densities (7 d after infusion, *p* = 0.039), the level of Ncan (*p* < 0.001), and the expression of PV (*p* = 0.002, Fig 6C). No significant effect was observed on Acan. These findings indicate that reduced Tgfβ2 expression occurring between early and late adolescence in RSP, driven at least in part by epigenetic changes of the *Tgfb2* gene, could contribute to the observed decline of PV, select lecticans and, consequently, decrease of PNN densities.

## Discussion

We demonstrated that RSP undergoes extensive molecular, cellular, and structural modifications during late adolescence that support the formation of “adult type” (long-lasting, context-specific) memories while interfering with the expression and fidelity of memories acquired during early adolescence. These findings identify a hitherto unrecognized, late maturation period of the RSP memory circuit with important consequences on memory dynamics.

While many studies have considered p60 mice as adult, neurobiological studies have shown that many processes related to brain development are still ongoing, although at a slower rate, beyond p60 [45,54,55]. To account for maturational differences related to inter-sexual, interindividual, and inter-strain differences in maturation, and for possible delays due to environmental and social factors, it was proposed that 3 months of age needs to be considered as the onset of adulthood [56]. For similar reasons (to reflect accumulating evidence of brain development into the early to mid-20s), the human development field has recently revised the standard view of human adolescence, changing the criteria from the age of 10–19 years to 10–24 years [57,58], a position also currently held by the NIH (“The brain finishes developing and maturing in the mid-to-late 20s.”) [59].

By showing that substantial cortical reorganization in mice taking place between p30 and p75 (which maps perfectly onto the human age of 10–24 years), and that there is a high variability in memory retention during this period, our findings are well-aligned with the revised views of developmental staging based on neurobiological criteria. Therefore, we consider the age between p60 and p75 as late adolescence in this study (green timeline in S1A Fig). Together with evidence that cortical myelination, which is essential for memory preservation [60], remains incomplete and continues beyond p60 [45,61,62], our findings indicate that PNN build up, another major processes underlying memory persistence [22,27,63], shows substantial instability during late adolescence affecting the retention of previously formed memories. These findings challenge the view that cortico-hippocampal memory circuits are fully mature by the age of p30 [31,32], and highlight the need to extend the studies of memory ontogeny and maturity beyond ages previously considered to reflect adulthood (i.e., p60).

Similar to our RSP findings, a decrease of PV and PNNs between p28 and p56 was previously reported for the dorsal intermediate entorhinal cortex, but not temporal cortical areas [64] or the medial prefrontal cortex [65], indicating regional differences of cortical maturation during adolescence. Indeed, a late adolescent reorganization (p60) of the mouse prefrontal cortex was recently reported, however, it was predominantly microglia-driven [48], whereas the RSP reorganization shown here was primarily PNN/PV interneuron-driven. Despite these mechanistic differences, however, both studies provided evidence for nonlinear cortical reorganization (increased initial maturation/activity during early adolescence followed by destabilization during late adolescence), which underlined the establishment of adult memory phenotypes.

In line with observations that remote memories are more sensitive to PNN loss than recent memories [22,63,66,67], impaired expression of context memories acquired at p30 was the strongest at p75, as they became remote, despite the fact that PNN levels were already reduced between p45 and p60. This could be due to hippocampal or other mechanisms compensating for RSP PNN degradation before memories become mainly cortically dependent.

As most previous rodent studies [30,31,68,69], ours showed no evidence of memory suppression [70]. However, the unreliable retrieval of early relative to late adolescent memories is at odds with the widely held view that memories formed during early adolescence are stable [31, 32]. An important reason for such discrepancy is that this view stems from hippocampal research, which does not take into consideration cortical control over memory expression as memories become less hippocampal- and more cortical-dependent with the passage of time [36,71–73]. Given that the decrease of RSP PNN densities paralleled the increase of DH PNN densities, we suspected that impairments of early adolescent memories might be triggered by the maturation of excitatory DH-RSP connections, establishing dominance of DH control over memory [33,74,75]. However, permanent inactivation of synaptic transmission in DH-RSP projections did not prevent the adolescent memory impairments, suggesting that the observed changes were more likely intrinsic to RSP rather than circuit driven, as previously found with PNN dynamics in the visual cortex [76,77].

We identified several RSP-intrinsic molecular mechanisms that could contribute to the observed PNN decline, including decreased levels of several key PNN constituents as well as reduced Tgfβ2 mRNA expression potentially linked to epigenetic modifications of the Tgfβ2 gene. In line with its demonstrated role in lectican gene expression, Tgfβ2 infusions increased the levels of neurocan and PNNs. Furthermore, the ECM stabilizer HAPLN1 also acts as an activator of the Tgfβ pathway [53,78,79], suggesting that Tgfβ could also mediate some of the effects of HAPLN1 in preserving PNNs and memory instability.

The memory expression deficits were not sensitive to the expression levels of PNNs or PV at a single time point, but to their decline between the time they were formed (early adolescence) and the time of retrieval tests (late adolescence). Accordingly, memories formed during late adolescence (p60–p75) were fully preserved in later life, although PNN and PV levels were lower relative to early adolescence. These findings can be explained by the contributions of PNN stabilization to the persistence of existing memories [22,27,63], and PNN destabilization to the formation of new memories [24,80]. Thus, the PNN degradation found in late adolescence could shift this balance towards the formation of new context memories, promoting adaptation to recent rather than remote contextual experiences, an adaptive process proposed for forgetting more generally [81,82].

The generalization of early adolescent context memories following fluctuations of their expression (impairment followed by spontaneous recovery) was consistent with the model of generalization rooted in memory instability [83], and indicated that memories acquired before late adolescence can still shape behavior in later life. An interesting possibility is that generalization might be related to the decreased overlap between PNNs and PV interneurons with the age, which could lie in the greater fluctuations of PV levels between adulthood and middle adulthood [84], in the progressive build-up around excitatory cortical neurons [85,86], or in other mechanisms.

Our findings suggest a more subtle contribution of endogenous PNN fluctuations to memory functions relative to exogenously applied enzymatic PNN and ECM degradation [23]. We propose two potential mechanisms by which such fluctuations could impair memory expression. One is a temporary failure to reconstruct the memory-related neuronal activity patterns. Alternatively (or additionally), RSP PNN fluctuations could dynamically prioritize or restrict access to memories based on their recency, valence, or other features. Either scenario would support a view of memory as a reconstructive process [87,88], a view that is gaining increasing support [89–92].

Some of our key behavioral findings are well-aligned with human studies. For example, the memory loss found in late adolescence is in line with the decrease of the associative structure of episodic memories between early and late adolescence [93], whereas the memory recovery shows striking similarities with the “reminiscence bump” phenomenon in human episodic memory research, which refers to the tendency of adults to recall a disproportionately large number of memories from their adolescence and early adulthood [94,95]. Also, the retention of the stress-related memory component (revealed by freezing behavior) but loss of context specificity, parallels the ease of remembering emotional memory content but impaired memory for contextual detail in human reminiscence bump [96]. Thus, an ongoing ECM reorganization in posterior cortices might also contribute to the human memory dynamics. As a downside, abnormalities of RSP ECM dynamics, especially during late adolescence, could increase disease risk of adult-onset mental illnesses, such as schizophrenia and major depression, especially in carriers of high-risk ECM gene variants [97], and contribute to the ECM abnormalities [98–100], contextual episodic memory deficits [101], and default mode network dysfunctions [102], reported for these illnesses.

There are several limitations of our study that remain to be addressed in future. We use PV immunoreactivity to determine putative PV interneuron presence. PV expressions are dynamically regulated, and reduction of its levels cannot be easily interpreted with respect to the interneuron number. The consistent use of reporters such as PV-Cre mice for longitudinal tracking of PV interneuron populations will help overcome confounds by variable PV levels and delineate clearly between PV loss and interneuron loss. While well-powered to identify general sex differences, our study was not sufficiently powered to identify effects of the estrous cycle, so we could have overlooked differences in memory retention or PNN dynamics related to the hormonal cycle of females. Our study focused on developmental changes in PNNs over a relatively long timeframe. However, short-term fluctuations in PNNs, including diurnal changes, have also been reported [103,104]. Therefore, future studies examining these short-term dynamics could help clarify their potential role during specific phases of memory formation. The role of PNNs varies across brain regions, cell populations, and memory types [23]. For example, PNNs surrounding CA2 pyramidal neurons have been implicated in social memory [105]. While supporting the role of RSP PNNs in adolescent CFC, our data does not rule out their contributions of other brain circuits and cell types relevant to CFC or other types of memories. Lastly, although we identified several epigenetic modifications of TGFBR2, the direct link between these modifications, PNN degradation, and memory retention, remain to be established.

## Materials and methods

### Ethics statement

All procedures were approved by Northwestern University Animal Care and Use Committee (protocols IS00002463 and IS00003359) and Albert Einstein College of Medicine Animal Care and Use Committee (protocols 00001289 and 00001268) in compliance with US National Institutes of Health standards.

### Animals

Wild-type (C57BL/6N) male and female mice were obtained from Envigo (Lafayette, IN, USA). Pvalb-IRES-Cre (PC-Cre, B6.129P2-Pvalb^tm1(cre)Arbr^/J, #017320) [106] and Neurotensin-IRES-tauGFP (NT-GFP; strain *Nts*^*tm1Mom*^/MomJ, #006702) [107] mice were obtained from Jackson Laboratory (Bar Harbor, ME, USA) and bred in the institutional animal facility. Animals were maintained under 12 h:12 h light:dark cycle with lights on at 07:00, temperature 20–22 °C, humidity 30%–60% with ad libitum access to food and water.

### Contextual fear conditioning

Contextual fear conditioning (CFC) was performed in automated system (TSE Systems, Berlin, Germany or Med Associates, Fairfax, VT, USA). Mice were exposed to Context A (CtxA, rectangular shape, metal shock grid floor, cleaned with 70% ethanol before each test) for 3 min, followed by a foot shock (0.7 mA, 2 s). On indicated days of test, freezing level of mice to the context were measured in CtxA or Context B (CtxB, triangular shape, white plastic floor, cleaned with 1% acetic acid before each test) for 3 min. Mice were removed immediately after shock delivery or upon completion of the 3 min test period. In most experiments, mice were tested once a week until p60 or p75, except for a spontaneous recovery experiment, in which mice were tested once a month until p181.

### CFC discrimination/generalization

The contextual discrimination/generalization task was performed over 4 days to explicitly determine the ability of mice to discriminate between CtxA and CtxB. On day 1, the mice were allowed to explore CtxA and CtxB, each for 3 min. On day 2–4, mice were exposed to CtxA for 3 min followed by a tone (30 s, 75 dB SPL, 10 kHz, 200 ms pulse) and a foot shock (0.7 mA, 2 s, constant current) sequentially once each day. On the day of test, freezing levels were measured during exposure of mice to CtxA and CtxB for 3 min each. The same tone used on day 2–4 was played at the end of CtxB test for 30 s to measure the freezing level to the auditory cue. Exposures to CtxA and CtxB were always spaced 3 h apart and counterbalanced. To alleviate the confounding effects of generalization found with the passage of time among similar contexts [108], we used dissimilar contexts that did not show time-dependent generalization (see S4 Fig).

### CFC reinstatement

Memory reinstatement was determined by measuring the effect of early adolescent CtxA CFC on late adolescence CtxB CFC. We subjected the mice to CFC in CtxA on p29 followed by 2 tests on p30 and p74. On p75 they were exposed to CtxB (as previously described, except the floor was replaced with shock grid) for 3 min and received a shock reminder (0.7 mA, 2 s) at the end of the exposure. On p76 freezing was determined in CtxA and CtxB for 3 min each, spaced 3 h apart, and counterbalanced. Interference of prior CFC in CtxA with freezing to CtxB and generalization of freezing to CtxA after CFC in CtxB served as indices of CtxA memory retention.

### Stereotaxic intracranial injection

Mice were anesthetized with isoflurane and placed on a stereotaxic instrument (Model 1900, Kopf Instruments, Tujunga, CA, USA). Viral vectors carrying AAV8-hSyn-mCherry (114472-AAV8, Addgene, gift from Karl Deisseroth), AAV8-hSyn-DIO-mCherry (50459-AAV8, Addgene, gift from Bryan Roth), AAV8/rg-hSyn-Cre-dTomato (107738-AAV8/rg, Addgene, gift from Rylan Larsen), AAVDJ-CMV-DIO-eGFP (GVVC-AAV-12, Stanford University Gene Vector and Virus Core), or AAVDJ-CMV-DIO-eGFP-2A-TeNT (Cre-Dependent Tetanus Toxin, GVVC-AAV-71, Stanford University Gene Vector and Virus Core) is delivered bilaterally into DH (1.8 mm posterior, 1.2 mm lateral, 2.25 mm ventral to bregma) and/or RSP (1.8 mm posterior, 1.2 mm lateral, 1.1 mm ventral to bregma) using a Hamilton microsyringe connected with microinfusing pump (UMP-3, WPI, Sarasota, FL, USA). For each site, 0.5 μL AAV solution (2 × 10^12^ GC/mL) was delivered at 0.15 μL/min, and syringes were left in place for 5 min after the injection. Recombinant HAPLN1 (2608-HP-025, R&D Systems, Minneapolis, MN, USA, 100 ng/μL in PBS), Tgfβ2 (7346-B2-005, R&D Systems, 10 ng/μL in PBS) was injected bilaterally into RSP (0.5 μL/site) as described above.

### Immunohistochemistry

Mice were anesthetized with an intraperitoneal injection of 240 mg/kg Avertin and transcardially perfused with ice-cold phosphate buffer followed by 4% paraformaldehyde in phosphate buffer (pH 7.4, 50 mL each per mouse). Brains were removed and post-fixed for 24 hours in the same fixative and then cryoprotected by immersing in 20% and 30% sucrose in phosphate buffer for 24 hours each. After that the brains were frozen in OCT embedding medium and 50 µm freezing microtome sections were made for use in free-floating lectin histochemistry or immunohistochemistry. Biotinylated WFA (1:1,000 B-1355–2, Vector Laboratories, Newark, CA, USA) was used to visualize PNNs. For immunohistochemistry, primary antibodies against aggrecan (1:1,000, ab3778, Abcam, Cambridge, UK), neurocan (1:2,000, AF5800 R&D systems), brevican (1:500, 19017-1-AP, Proteintech, Rosemont, IL, USA), phosphacan (1:500, sc-33664, Santa Cruz, Dallas, TX, USA), PV (1:5,000, pvg-213, Swant, Burgdorf, Switzerland), SST (1:1,000, MAB354, Millipore, Burlington, MA, USA), VIP (1:1,000, 20077-1513001, Immunostar, Hudson, WI, USA), NPY (1:1000, 22940-1628001, Immunostar), CR (1:4,000, CG1, Swant), Iba1 (1:1,000, ab5076, Abcam), Olig2 (1:2,000, ab109186, Abcam), PDGFRa (1:2,000, AF1062, R&D Systems), and s100b (1:2,000, 287108, Synaptic Systems, Goettingen, Germany) were used followed by corresponding secondary antibodies (1:500, Alexa Fluor conjugated, Jackson IR, West Grove, PA, USA or 1:200, Biotinylated, Vector Laboratories). When using biotinylated systems, the signals were visualized with the ABC-HRP Detection Kit (PK-6100, Vector Laboratories) and TSA Fluorophores (Akoya Biosciences, Marlborough, MA, USA).

### Microscopy, image analysis and quantification

For large scale analyses, sections were scanned using Axioscan Z1 microscope (Zeiss, Oberkochen, Germany) scanner under 10×/0.45 air objective. Images were stitched and converted to TIFF format. RSP findings were additionally verified following confocal microscopy under 60×/1.35 oil objective (Fluoview FV10i-LIV, Olympus, Hachioji, Tokyo, Japan). For each experiment, the same acquisition profile, including camera and excitation settings, was used across all experimental groups. All quantifications were performed with ImageJ using two methods: the multi point plug-in and analyze particles plug-in. Intensity was calculated by measuring the gray value after thresholding to eliminate background interference. Cell counting and gray value analysis were performed in a 0.1 mm^2^ area for each ROIs. The identical threshold was applied across experimental groups. Two sections per animal were collected and the measurements were averaged. Each animal was treated as an independent sample for statistical analysis.

Volume analyses were performed within targeted RSP region starts at the first appearance of the suprapyramidal blade granular layer cells and ends at the first appearance of subcommissural organ (anterior-posterior direction, coronal plates). ADMBA p28 and p56 coronal Nissl datasets were downloaded using Allen API. Images were inspected and the distance between end plates is obtained from the metadata. Areas of RSP were measured using ImageJ at 200 µm intervals to calculate the volume. DeMBA volume and segmentation files (CCFv3, 2022 edition) were downloaded from ebrains.eu. Data were visualized with ITK-SNAP [109] to determine range of z-plates. RSP voxels within the range were counted and converted to volumes using NiBabel [110] python package.

### Quantitative PCR analysis

Mice subjected to fresh tissue collection were euthanized with cervical dislocation. After that, the brain was removed and DH and RSP tissue were dissected from the brain. Total RNA was extracted using RNeasy Plus Mini Kit (74136, Qiagen). Reverse transcription was performed on 100 ng of total RNA using PrimeScript RT Reagent Kit (RR037A, Takara, Kusatsu City, Japan). Quantitative PCR analysis was performed on a QuantStudio 6 Flex instrument (Applied Biosystems, Waltham, MA, USA) using SYBR green detection system (4367659, Applied Biosystems) and primers specific for *Tgfb1* (QT00145250, Qiagen), *Tgfb2* (QT00106806, Qiagen), and *Tgfbr2* (QT00135646, Qiagen). GAPDH (F: AACTTTGGCATTGTGGAAGG; R: ACACATGGGGGTAGGAACA) was used as endogenous control. mRNA levels were calculated using the comparative CT method (2^−ΔΔCt^).

### DNA methylation assay

DNA was extracted using the Qiagen AllPrep DNA/RNA/miRNA Universal Kit (Qiagen; Hilden, Germany; cat# 80224) per the manufacturer’s protocol. 500 ng of extracted DNA samples were bisulfite modified using the EZ-96 DNA Methylation Kit (ZymoResearch; Irvine, CA; cat# D5004). After that, all bisulfite-modified DNA samples were amplified using separate multiplex or simplex PCRs. Samples were run alongside established reference DNA with 0%, 5%, 10%, 25%, 50%, 75%, and 100% methylation. Sequencing libraries were prepared using a custom library preparation method created by EpigenDx. Next, library molecules were purified using Agencourt AMPure XP beads (Beckman Coulter; Brea, CA; cat# A63882). Barcoded samples were then pooled in an equimolar fashion before template preparation and enrichment were performed on the Ion Chef system using Ion 520 and Ion 530 ExT Chef reagents (Thermo Fisher; Waltham, MA; cat# A30670). Following this, enriched, template-positive library molecules were sequenced on the Ion S5 sequencer using an Ion 530 sequencing chip (cat# A27764). For data analysis, FASTQ files from the Ion Torrent S5 server were aligned to a local reference database using the open-source Bismark Bisulfite Read Mapper program (v0.12.2) with the Bowtie2 alignment.

### Statistical analysis

Statistical power to detect anticipated effect sizes was determined using power analysis (calculator at http://www.stat.ubc.ca/~rollin/stats/ssize/n2.html) conducted on representative samples of previous work and pilot experiments. For all proposed experiments, minimum power is set at 0.90 to detect an *α* = 0.05 (two-sided test) for a difference in means from 20% to 40%, with a 15% common standard deviation. Mice are randomly assigned to groups. However, to prevent litter effects, mice from the same litter were assigned to different experimental groups. Viruses and proteins were injected by experimenters aware of the construct, but the mice were then assigned coded numbers by the laboratory technician. The code was available after quantification and before analyses. Statistical analyses were performed using GraphPad Prism. Outlier mice exhibiting abnormal behavior performance determined by Grubbs’ test or with misplaced infusion were excluded. One-way ANOVA or Two-way ANOVA followed by indicated post hoc tests were used for comparisons of three or more experimental groups (only when ANOVA was significant) whereas Student *t* test was used for comparison of two experimental groups. Homogeneity of variance was confirmed with Levene’s test for equality of variances. Repeated measures (RM) ANOVAs with Geisser Greenhouse correction were performed for within-subject designs. On indicated data, we performed correlation analyses and report Pearson’s r coefficients.

All comparisons were conducted using two-tailed tests and the *P* value for all cases was set to <0.05 for significant differences. Data are expressed as mean ± s.e.m. Statistically significant differences are indicated as *p < 0.05, **p < 0.01, and ***p < 0.001.

## Supporting information

S1 Fig PNN changes throughout adolescence by region and sex. **(A)** Left: The standard view that human adolescence encompasses a period from roughly the age of 10–19 years (yellow) was recently revised to 10–24 years (green) to reflect changes of biological growth and other factors [57,58]. The approximate human-mouse age alignment was calculated according to Dutta and colleagues [54]. The experimental design and nomenclature were aligned with the later view; right: representative images of p21, p30, p45, p60, and p75 male brain sections stained with WFA. **(B)** Representative images for WFA staining in RSP (top, males) and quantification for the density of PNN^+^ cells demonstrating significant post-adolescent decreases in the density of PNNs (males bottom left; p21: *n* = 3, p30 and p75: *n* = 5, p45 and p60: *n* = 4; one-way ANOVA: *F* = 8.124, *p* < 0.001; Tukey’s post-hoc test: p30 vs. p45: *p* = 0.002, p30 vs. p60: *p* = 0.002; females bottom right, p21: *n* = 3, p60: *n* = 4, others: *n* = 5; one-way ANOVA: *F* = 9.032, *p* < 0.001; Tukey’s post-hoc test: p30 vs. p45: *p* < 0.001, p30 vs. p60: *p* = 0.002) RSP. **(C)** Representative images for WFA staining in SUB (top, males) and quantification for the density of PNN^+^ cells revealing similar decreases in SUB (males bottom left; p21: *n* = 3, p30: *n* = 5, others: *n* = 4; one-way ANOVA: *F* = 3.197, *p* = 0.044; Tukey’s post-hoc test: p30 vs. p75: *p* = 0.025; females bottom right, p21: *n* = 3, p60: *n* = 4, others: *n* = 5; one-way ANOVA: *F* = 3.429, *p* = 0.031; Tukey’s post-hoc test: p30 vs. p60: *p* = 0.034) SUB. **(D)** Representative images for WFA staining in DH (top, males) and quantification for the density of PNN^+^ cells showing stable or increased density of PNNs (males bottom left; p21: *n* = 3, p30 and p75: *n* = 5, p45 and p60: *n* = 4; two-way RM ANOVA: Region: *F*_(1.905,30.48)_ = 95.74 *p* < 0.001, Age: *F*_(4, 16)_ = 3.533 *p* = 0.030, Region × Age: *F*_(12,48)_ = 1.273 *p* = 0.265; Tukey’s post-hoc test: CA3: p21 vs. p75: *p* = 0.048, DG: p21 vs. p45: *p* = 0.035, p21 vs. p60: *p* = 0.003, p21 vs. p75: *p* < 0.001, p30 vs. p75: *p* = 0.036, p45 vs. p75: *p* = 0.048, p60 vs. p75: *p* = 0.020; females bottom right, p21: *n* = 3, p60: *n* = 4, others: *n* = 5; two-way RM ANOVA: Region: *F*_(1.893,32.17)_ = 149.1 *p* < 0.001, Age: *F*_(4, 17)_ = 7.984 *p* < 0.001, Region × Age: *F*_(12,51)_ = 0.928 *p* = 0.527; Tukey’s post-hoc test: CA1: p21 vs. p60: *p* = 0.019, CA3: p60 vs. p75: *p* = 0.031) CA1, CA2, CA3, and DG. Data represent mean ± s.e.m., ***p < 0.001; **p < 0.01; *p < 0.05. The data underlying this Figure can be found in S1 Data.
(TIF)

S2 Fig Developmental changes of glial cells in RSP. Left: Density of microglia, astrocyte, oligodendrocyte precursor cell (OPC), and oligodendrocyte showing an increase for astrocyte and oligodendrocyte (*n* = 6; microglia: one-way ANOVA: *F* = 0.006640, *p* = 0.936; astrocyte: one-way ANOVA: *F* = 6.658, *p* = 0.009, Tukey’s post-hoc test: p30 vs. p60: *p* = 0.023, p30 vs. p75: *p* = 0.013; OPC: one-way ANOVA: *F* = 1.018, *p* = 0.385; oligodendrocyte: bottom right, *F* = 32.33, *p* < 0.001, Tukey’s post-hoc test: p30 vs. all: *p* < 0.001, p60 vs. p75: *p* = 0.024); right: representative images. The data underlying this Figure can be found in S1 Data.
(TIF)

S3 Fig Context specificity of CFC memories acquired during early adolescence (p25–p35). Context discrimination task (top) shows that mice were able to form specific contextual fear memory after p28 (*n* = 9/age group and sex, bottom left, three-way RM ANOVA, Sex: *F*_(1,62)_ = 1.413, *p* = 0.239, Age: *F*_(3,62)_ = 0.7597, *p* = 0.521, Ctx: *F*_(1,62)_ = 588.2, *p* < 0.001, Age × Sex: *F*_(3,62)_ = 0.7293, *p* = 0.538, Age × Ctx: *F*_(3,62)_ = 34.50, *p* < 0.001, Sex × Ctx: *F*_(1,62)_ = 5.748, *p* = 0.020, Age × Sex × Ctx: *F*_(3,62)_ = 5.065, *p* = 0.003, Tukey’s post-hoc test: Ctx A vs. Ctx B in all groups other than p25 male and p25 female: *p* < 0.001). And that they show normal fear response to auditory cue since p21 (p32 male and p48 female: *n* = 8, other: *n* = 9, bottom right, two-way ANOVA: Age × Sex: *F*_(3,62)_ = 1.247, *p* = 0.300, Age: *F*_(3,62)_ = 1.069, *p* = 0.369, Sex: *F*_(1,62)_ = 0.3027, *p* = 0.584). Experimental diagrams were created in BioRender. Zhang, H. (2026) https://BioRender.com/kz4bcow. Data represent mean ± s.e.m., ***p < 0.001. The data underlying this Figure can be found in S1 Data.
(TIF)

S4 Fig Specificity of recent and remote contextual fear memory formed during late adolescence (p60–p63). Contextual discrimination task performed on p60 mice shows a prolonged memory specificity in distinguishing dissimilar contexts (same setup used in this study for p28 mice, without tone) up to p150 (two-way RM ANOVA: Age of test: *F*_(1.890, 18.90)_ = 2.209 *p* = 0.139, Context: *F*_(1, 10)_ = 370.2 *p* < 0.001, Age of test × Context: *F*_(1.897, 18.97)_ = 2.532 *p* = 0.108). Experimental diagrams were created in BioRender. Zhang, H. (2026) https://BioRender.com/qmq1etq. Data represent mean ± s.e.m., ***p < 0.001. The data underlying this Figure can be found in S1 Data.
(TIF)

S5 Fig Effect of inactivating DH to RSP projections during late adolescence on the retrieval of previously acquired memories. **(A)** Left, Delineation of brain areas showing strong NTS labeling in thalamic, hippocampal, and RSP regions during early development (p7–p14) in NTS-GFP mice [46]. Right, Representative images of NTS labeling showing two projections from SUB to RSP terminating in L2/3 and L5. **(B)** Disappearance of NTS from DH to RSP projections between p30 and p60. **(C)** Mature DH to RSP projections to RSP L1 and L2/3 (left), with excitatory projections terminating in L2/3 (middle) and lack of terminals in L5 (right). **(D)** Inhibition of DH to RSP projections does not affect the impaired retrieval of adolescent memories (*n* = 12, two-way RM ANOVA: Tettox: *F*_(1,21)_ = 0.1627 *p* = 0.691, Age of test: *F*_(1, 21)_ = 30.14 *p* < 0.001 Infusion × Age of test: *F*_(1,21)_ = 0.0040 *p* = 0.950; Šídák’s post-hoc test: Control p30 vs. p61: *p* = 0.002, Tettox p30 vs. p61: *p* = 0.002). Experimental diagrams were created in BioRender. Zhang, H. (2026) https://BioRender.com/m7zoc9h. Data represent mean ± s.e.m., **p < 0.01. The data underlying this Figure can be found in S1 Data.
(TIF)

S6 Fig Freezing behavior after shock re-exposure in a different context. **(A)** Left, Experimental paradigm; Right, Shock in a novel context (Context B, CtxB) on p75 resulted in Context B-specific freezing, but also in increased freezing to the conditioned context (Context A, CtxA) (One-way RM ANOVA, *F*_(2.431, 21.88)_ = 15.01, *p* < 0.001, Tukey’s post-hoc test: p30 vs. p60: *p* = 0.004, p30 vs. p74: *p* < 0.001, p44 vs. p74: *p* = 0.002, p60 vs. p74: *p* < 0.001, p74 vs. p76: *p* = 0.011). **(B)** The experiment was repeated with a no-shock control group, and no tests performed between p30 and p74. Shock in CtxB on p75 did not increase freezing in CtxA in no shock group(*n* = 17 for shock group, *n* = 12 for no shock group; two-way RM ANOVA: Test: *F*_(1.657, 44.75)_ = 10.22, *p* < 0.001, Shock: *F*_(1, 27)_ = 51.12, *p* < 0.001, Test × Shock: *F*_(1.657, 44.75)_ = 15.35, *p* < 0.001; Tukey’s post-hoc test: shock group: p30 vs. p74: *p* < 0.001, p30 vs. p76: *p* = 0.041 p74 vs. p76: *p* = 0.004, no shock group: p30 vs. p76: *p* = 0.008, shock vs. no shock: p30: *p* < 0.001, p76: *p* = 0.005). Experimental diagrams were created in BioRender. Zhang, H. (2026) https://BioRender.com/wgbkg4j. Data represent mean ± s.e.m. ***p < 0.001; **p < 0.01; *p < 0.05. Mice that shocked on p30 and exhibited less than a 20% decline in freezing at p75 relative to p30 were excluded. See Fig 3C for data without this exclusion. The data underlying this Figure can be found in S1 Data.
(TIF)

S7 Fig Spontaneous recovery of early adolescent contextual fear memory and change of PNN-interneuron association in RSP by p150. **(A)** Spontaneous recovery of adolescent aversive context memory by mid-adulthood/p150 (*n* = 16; one-way RM ANOVA: *F* = 8.025, *p* < 0.001; Tukey’s post-hoc test: p30 vs. p90: *p* < 0.001, p90 vs. p150: *p* = 0.002, p90 vs. p180: *p* = 0.012). **(B)** Increased proportion of RSP PNN-free PV interneuron in p150 compared to p30 (*n* = 6; unpaired two-tailed *t* test: *t*_10_ = 8.539, *p* < 0.001). **(C)** Decreased co-localization between PV and PNNs was not due to increased PNN formation around other interneuron classes, which showed a significant lack of co-localization with WFA relative to PV neurons at p30 and p150 (two-way ANOVA: Interneuron type: *F*_(4, 50)_ = 560.8, *p* < 0.001 Age: *F*_(1, 50)_ = 33.53, *p* < 0.001, Interaction: *F*_(4, 50)_ = 39.44, *p* < 0.001, Šídák’s post-hoc test: p30 vs. p150: PV: *p* < 0.001, SST: *p* = 0.999, VIP: *p* = 0.690, NPY: *p* > 0.999, CR: *p* > 0.999). Experimental diagrams were created in BioRender. Zhang, H. (2026) https://BioRender.com/9yzhxu7. Data represent mean ± s.e.m. ***p < 0.001; **p < 0.01; *p < 0.05. The data underlying this Figure can be found in S1 Data.
(TIF)

S8 Fig Levels and developmental changes of phosphacan across RSP layers. **(A)** Low magnification images demonstrating the cortical, hippocampal, and thalamic distribution of phosphacan and WFA at p21 (males *n* = 4, females *n* = 3), p30 (males, *n* = 5, females *n* = 5), and p75 (males *n* = 4, females *n* = 4). At p30, the highest levels are obtained in layer 1, containing the cortical axons. **(B)** High magnification images showing progressive post-adolescent down-regulation of phosphacan across layers and ECM compartments including but not restricted to PNNs. **(C)** Layer-by-layer quantification of phosphacan optical density demonstrating significant effects in males (two-way ANOVA: Age: *F*_(2, 50)_ = 42.32, *p* < 0.001, Layer: *F*_(4, 50)_ = 17.70, *p* < 0.001, Age × Layer: *F*_(8, 50)_ = 6.574, *p* < 0.001, Tukey’s post-hoc test: p21 vs. p30: *p* < 0.001, p21 vs. p75: *p* < 0.001, p30 vs. p75: *p* < 0.001) and females (two-way ANOVA: Age: *F*_(2,45)_ = 12.36, *p* < 0.001, Layer: *F*_(4,45)_ = 1.478, *p* = 0.225, Age × Layer: *F*_(8,45)_ = 0.5702, *p* = 0.797, Tukey’s post-hoc test: p21 vs. p30: *p* < 0.001, p21 vs. p75: *p* < 0.001). Data represent mean ± s.e.m., ***p < 0.001; **p < 0.01; *p < 0.05. The data underlying this Figure can be found in S1 Data.
(TIF)

S9 Fig Post-adolescent levels of PNN-associated lecticans in DH and SUB. **(A)** Developmental decreases of PNN composition in DH (male aggrecan: p21: *n* = 4, other: *n* = 5, two-way ANOVA: Age: *F*_(2, 44)_ = 12.14, *p* < 0.001, Area: *F*_(3, 44)_ = 15.71, *p* < 0.001, Age × Area: *F*_(6, 44)_ = 3.146, *p* = 0.012, Tukey’s post-hoc test: p21 vs. p30: *p* < 0.001, p21 vs. p75: *p* = 0.011; female aggrecan: p21: *n* = 3, other: *n* = 5, two-way ANOVA: Age: *F*_(2, 40)_ = 3.515, *p* = 0.039, Area: *F*_(3, 40)_ = 14.21, *p* < 0.001, Age × Area: *F*_(6, 40)_ = 1.438, *p* = 0.225; male neurocan: p21: *n* = 3, other: *n* = 5, two-way ANOVA: Age: *F*_(2, 40)_ = 9.519, *p* < 0.001, Area: *F*_(3, 40)_ = 17.99, *p* < 0.001, Age × Area: *F*_(6, 40)_ = 0.2076, *p* = 0.972, Tukey’s post-hoc test: p21 vs. p30: *p* = 0.027, p21 vs. p75: *p* < 0.001; female neurocan: p21: *n* = 3, other: *n* = 5, two-way ANOVA: Age: *F*_(2, 40)_ = 2.812, *p* = 0.072, Area: *F*_(3, 40)_ = 16.57, *p* < 0.001, Age × Area: *F*_(6, 40)_ = 0.7006, *p* = 0.651; male brevican: p30: *n* = 5, other: *n* = 4, two-way ANOVA: Age: *F*_(2, 40)_ = 1.567, *p* = 0.221, Area: *F*_(3, 40)_ = 15.22, *p* < 0.001, Age × Area: *F*_(6, 40)_ = 1.302, *p* = 0.279; female brevican: p21: *n* = 2, p30: *n* = 4, p75: *n* = 5, two-way ANOVA: Age: *F*_(2, 32)_ = 15.37, *p* < 0.001, Area: *F*_(3, 32)_ = 4.618, *p* = 0.009, Age × Area: *F*_(6, 32)_ = 1.735, *p* = 0.145, Tukey’s post-hoc test: p21 vs. p30: *p* = 0.006, p21 vs. p75: *p* < 0.001, p30 vs. p75: *p* = 0.038; male phosphacan: p21: *n* = 4, other: *n* = 5, two-way ANOVA: Age: *F*_(2, 70)_ = 20.37, *p* < 0.001, Area: *F*_(6, 70)_ = 3.190, *p* = 0.008, Age × Area: *F*_(12, 70)_ = 1.307, *p* = 0.235, Tukey’s post-hoc test: p21 vs. p75: *p* < 0.001, p30 vs. p75: *p* < 0.001; female phosphacan: p21: *n* = 3, other: *n* = 5, two-way ANOVA: Age: *F*_(2, 70)_ = 7.449, *p* = 0.001, Area: *F*_(6, 70)_ = 5.615, *p* < 0.001, Age × Area: *F*_(12, 70)_ = 1.868, *p* = 0.054, Tukey’s post-hoc test: p21 vs. p75: *p* = 0.017, p30 vs. p75: *p* = 0.002). **(B)** Developmental change of PNN composition in SUB (male aggrecan: p30: *n* = 5, other: *n* = 4, one-way ANOVA: *F* = 5.746, *p* = 0.022; Tukey’s post-hoc test: p30 vs. p75: *p* = 0.017; female aggrecan: p21: *n* = 3, other: *n* = 5, one-way ANOVA: *F* = 124.9, *p* < 0.001; Tukey’s post-hoc test: p21 vs. p75: *p* < 0.001, p30 vs. p75: *p* < 0.001; male neurocan: p21: *n* = 3, p30: *n* = 5, p75: *n* = 4, one-way ANOVA: *F* = 4.951, *p* = 0.036; Tukey’s post-hoc test: p30 vs. p75: *p* = 0.047; female neurocan: p21: *n* = 3 other: *n* = 5, one-way ANOVA: *F* = 0.5794, *p* = 0.578; male brevican: p21: *n* = 3, p30: *n* = 5, p75: *n* = 4, one-way ANOVA: *F* = 0.2091, *p* = 0.815; female brevican: p21: *n* = 2, p30: *n* = 3, p75: *n* = 5, one-way ANOVA: *F* = 11.40, *p* = 0.006, Tukey’s post-hoc test: p21 vs. p75: *p* = 0.036, p30 vs. p75: *p* = 0.008; male phosphacan: p30: *n* = 5, other: *n* = 4, one-way ANOVA: *F* = 11.01, *p* = 0.003, Tukey’s post-hoc test: p21 vs. p75: *p* = 0.002; female phosphacan: p21: *n* = 3, other: *n* = 5, one-way ANOVA: *F* = 29.62, *p* < 0.001, Tukey’s post-hoc test: p21 vs. p30: *p* = 0.001, p21 vs. p75: *p* < 0.001, p30 vs. p75: *p* = 0.027). Data represent mean ± s.e.m., ***p < 0.001; **p < 0.01; *p < 0.05. The data underlying this Figure can be found in S1 Data.
(TIF)

S1 Data Data used for graphs and statistical analysis. (XLSX)

## Abbreviations

ADMBA: Allen Developing Mouse Brain Atlas
CFC: contextual fear conditioning
DeMBA: developmental mouse brain atlas
DH: dorsal hippocampus
ECM: extracellular matrix
GFP: green fluorescent protein
NTS: neurotensin
PNNs: perineuronal nets
PV: parvalbumin
RM: repeated measures
RSP: retrosplenial
SUB: subiculum
WFA: wisteria floribunda agglutinin
