# Supplementary figures and images for: Retrosplenial cortical reorganization during late adolescence introduces instability of contextual memory circuits
Source: PLoS Biol. 2026 Jul 17;24(7):e3003908. doi: 10.1371/journal.pbio.3003908 (PMC13378997; doi:10.1371/journal.pbio.3003908)

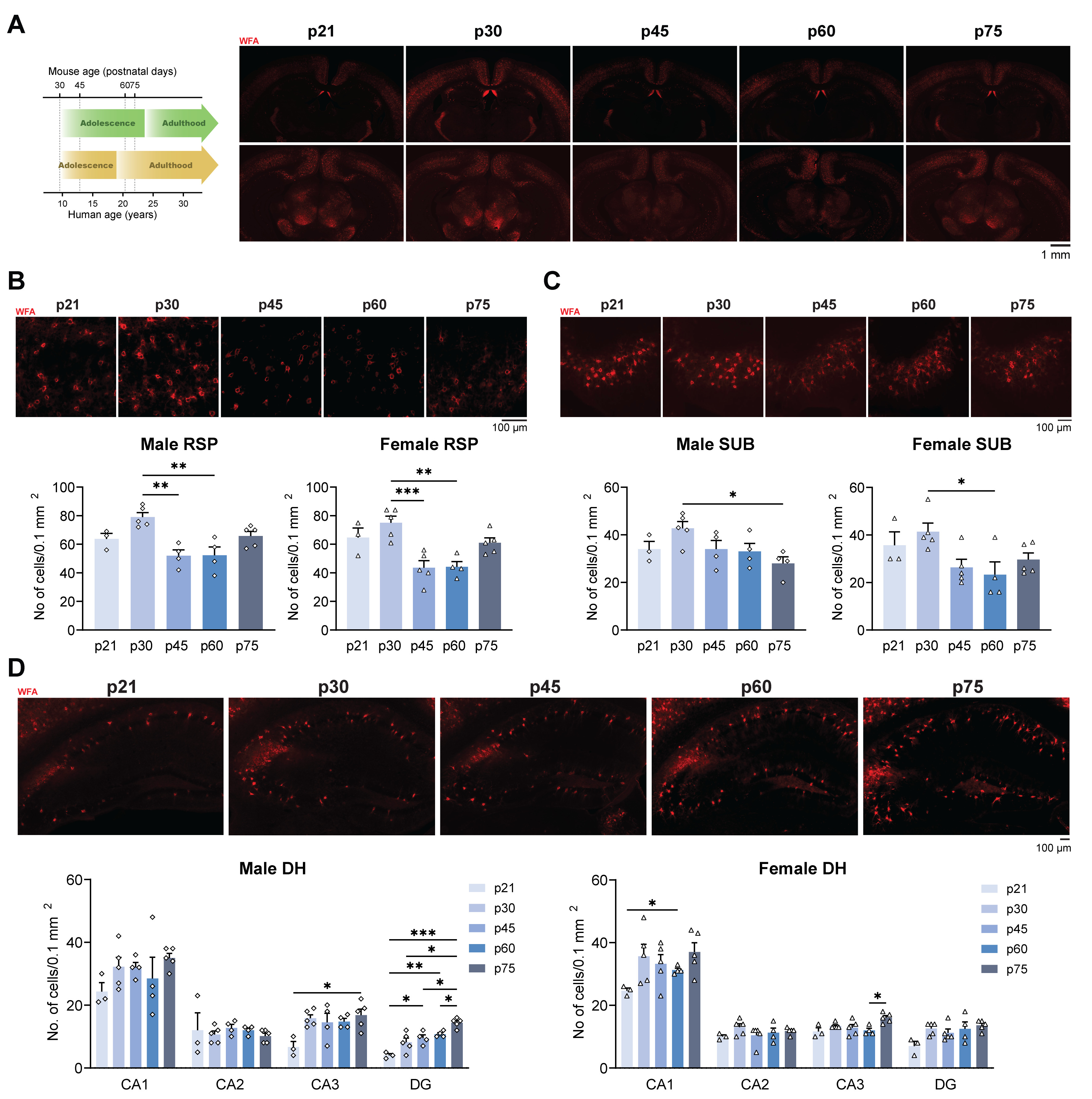

Supplement: S1 Fig — (A) Left: The standard view that human adolescence encompasses a period from roughly the age of 10–19 years (yellow) was recently revised to 10–24 years (green) to reflect changes of biological growth and other factors [57,58]. The approximate human-mouse age alignment was calculated according to Dutta and colleagues [54]. The experimental design and nomenclature were aligned with the later view; right: representative images of p21, p30, p45, p60, and p75 male brain sections stained with WFA. (B) Representative images for WFA staining in RSP (top, males) and quantification for the density of PNN+ cells demonstrating significant post-adolescent decreases in the density of PNNs (males bottom left; p21: n = 3, p30 and p75: n = 5, p45 and p60: n = 4; one-way ANOVA: F = 8.124, p < 0.001; Tukey’s post-hoc test: p30 vs. p45: p = 0.002, p30 vs. p60: p = 0.002; females bottom right, p21: n = 3, p60: n = 4, others: n = 5; one-way ANOVA: F = 9.032, p < 0.001; Tukey’s post-hoc test: p30 vs. p45: p < 0.001, p30 vs. p60: p = 0.002) RSP. (C) Representative images for WFA staining in SUB (top, males) and quantification for the density of PNN+ cells revealing similar decreases in SUB (males bottom left; p21: n = 3, p30: n = 5, others: n = 4; one-way ANOVA: F = 3.197, p = 0.044; Tukey’s post-hoc test: p30 vs. p75: p = 0.025; females bottom right, p21: n = 3, p60: n = 4, others: n = 5; one-way ANOVA: F = 3.429, p = 0.031; Tukey’s post-hoc test: p30 vs. p60: p = 0.034) SUB. (D) Representative images for WFA staining in DH (top, males) and quantification for the density of PNN+ cells showing stable or increased density of PNNs (males bottom left; p21: n = 3, p30 and p75: n = 5, p45 and p60: n = 4; two-way RM ANOVA: Region: F(1.905,30.48) = 95.74 p < 0.001, Age: F(4, 16) = 3.533 p = 0.030, Region × Age: F(12,48) = 1.273 p = 0.265; Tukey’s post-hoc test: CA3: p21 vs. p75: p = 0.048, DG: p21 vs. p45: p = 0.035, p21 vs. p60: p = 0.003, p21 vs. p75: p < 0.001, p30 vs. p75: p = 0.036, p45 vs. [file pbio.3003908.s001.tif]

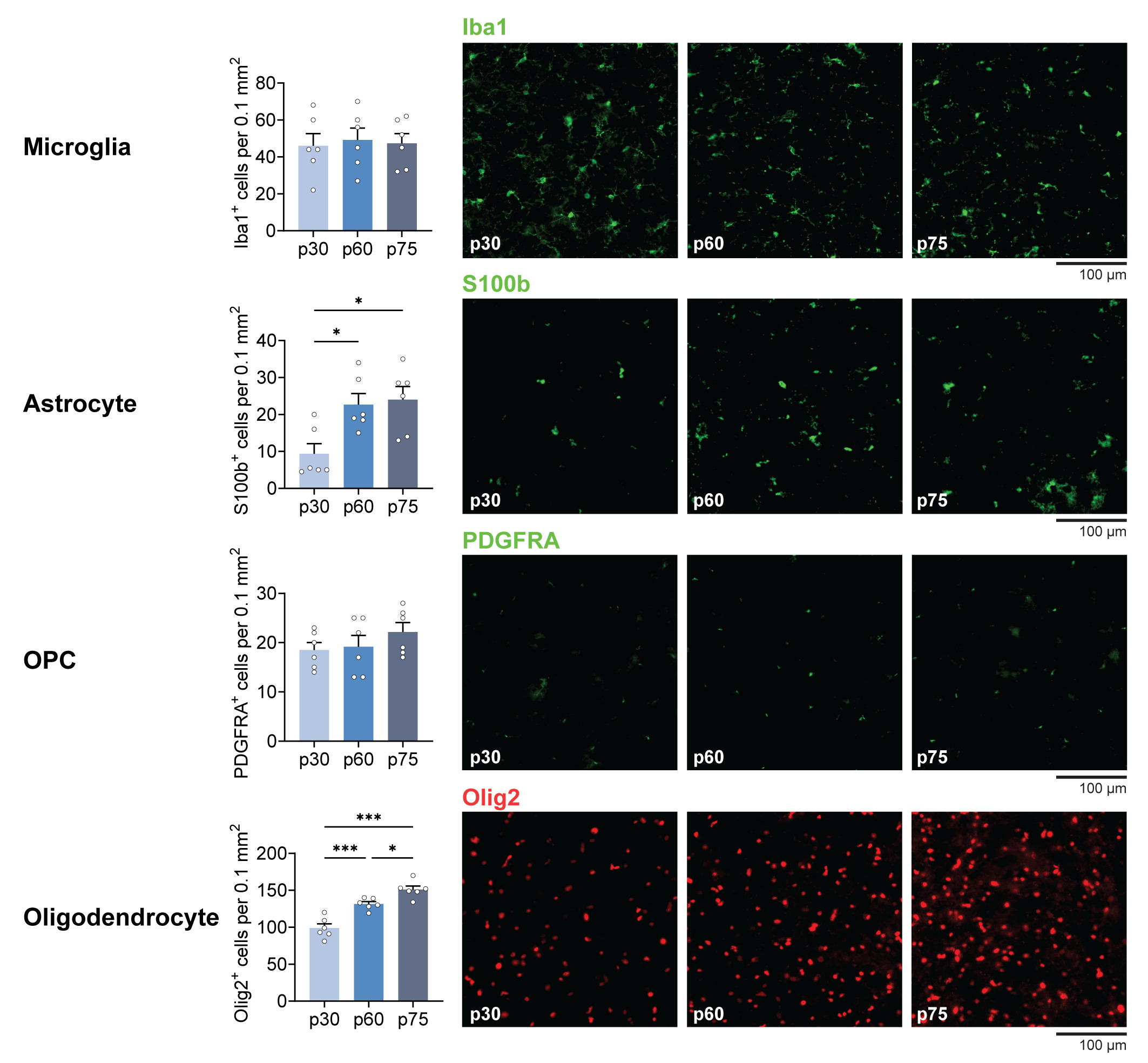

Supplement: S2 Fig — Left: Density of microglia, astrocyte, oligodendrocyte precursor cell (OPC), and oligodendrocyte showing an increase for astrocyte and oligodendrocyte (n = 6; microglia: one-way ANOVA: F = 0.006640, p = 0.936; astrocyte: one-way ANOVA: F = 6.658, p = 0.009, Tukey’s post-hoc test: p30 vs. p60: p = 0.023, p30 vs. p75: p = 0.013; OPC: one-way ANOVA: F = 1.018, p = 0.385; oligodendrocyte: bottom right, F = 32.33, p < 0.001, Tukey’s post-hoc test: p30 vs. all: p < 0.001, p60 vs. p75: p = 0.024); right: representative images. The data underlying this Figure can be found in S1 Data. (TIF) [file pbio.3003908.s002.tif]

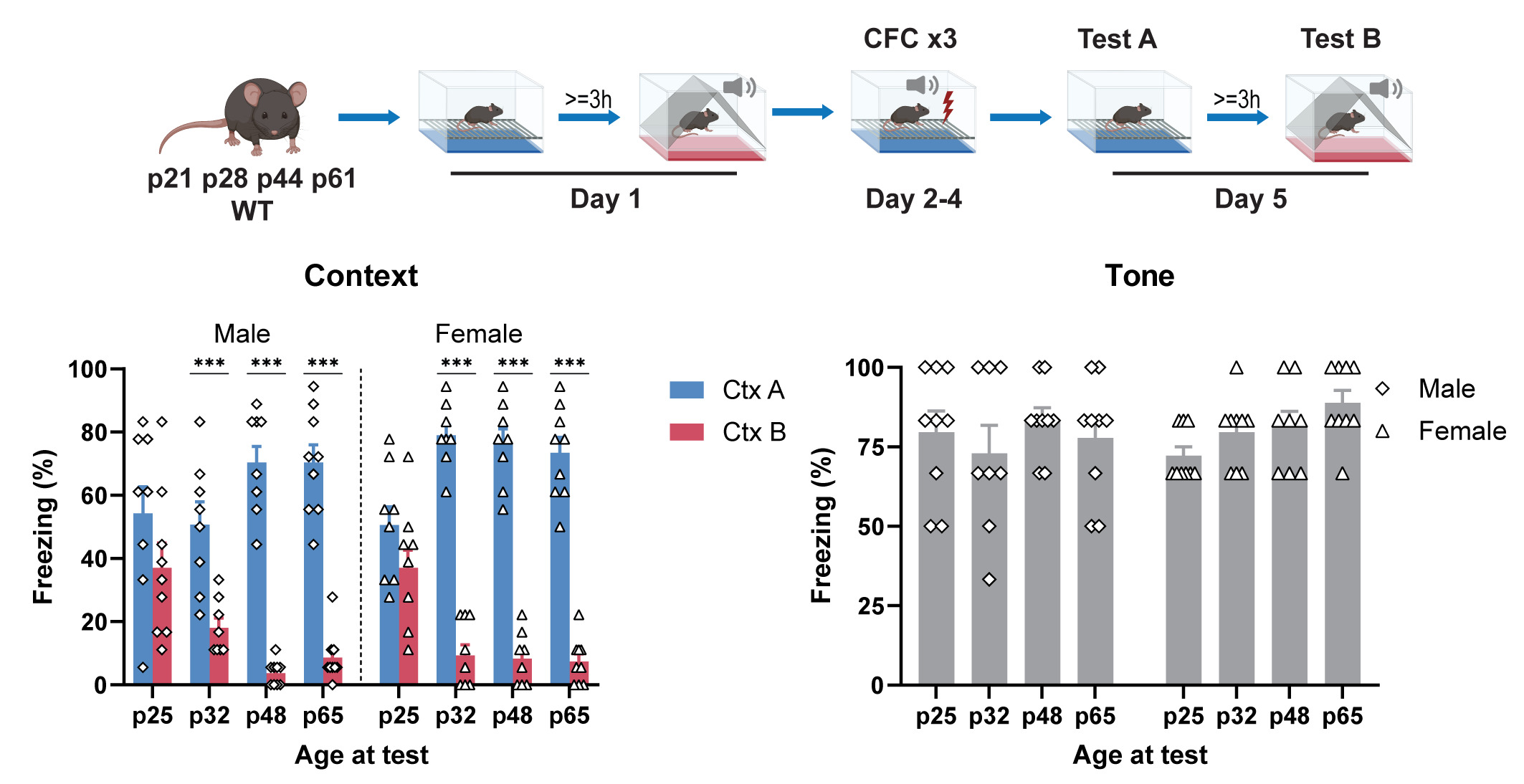

Supplement: S3 Fig — Context discrimination task (top) shows that mice were able to form specific contextual fear memory after p28 (n = 9/age group and sex, bottom left, three-way RM ANOVA, Sex: F(1,62) = 1.413, p = 0.239, Age: F(3,62) = 0.7597, p = 0.521, Ctx: F(1,62) = 588.2, p < 0.001, Age × Sex: F(3,62) = 0.7293, p = 0.538, Age × Ctx: F(3,62) = 34.50, p < 0.001, Sex × Ctx: F(1,62) = 5.748, p = 0.020, Age × Sex × Ctx: F(3,62) = 5.065, p = 0.003, Tukey’s post-hoc test: Ctx A vs. Ctx B in all groups other than p25 male and p25 female: p < 0.001). And that they show normal fear response to auditory cue since p21 (p32 male and p48 female: n = 8, other: n = 9, bottom right, two-way ANOVA: Age × Sex: F(3,62) = 1.247, p = 0.300, Age: F(3,62) = 1.069, p = 0.369, Sex: F(1,62) = 0.3027, p = 0.584). Experimental diagrams were created in BioRender. Zhang, H. (2026) https://BioRender.com/kz4bcow. Data represent mean ± s.e.m., ***p < 0.001. The data underlying this Figure can be found in S1 Data. (TIF) [file pbio.3003908.s003.tif]

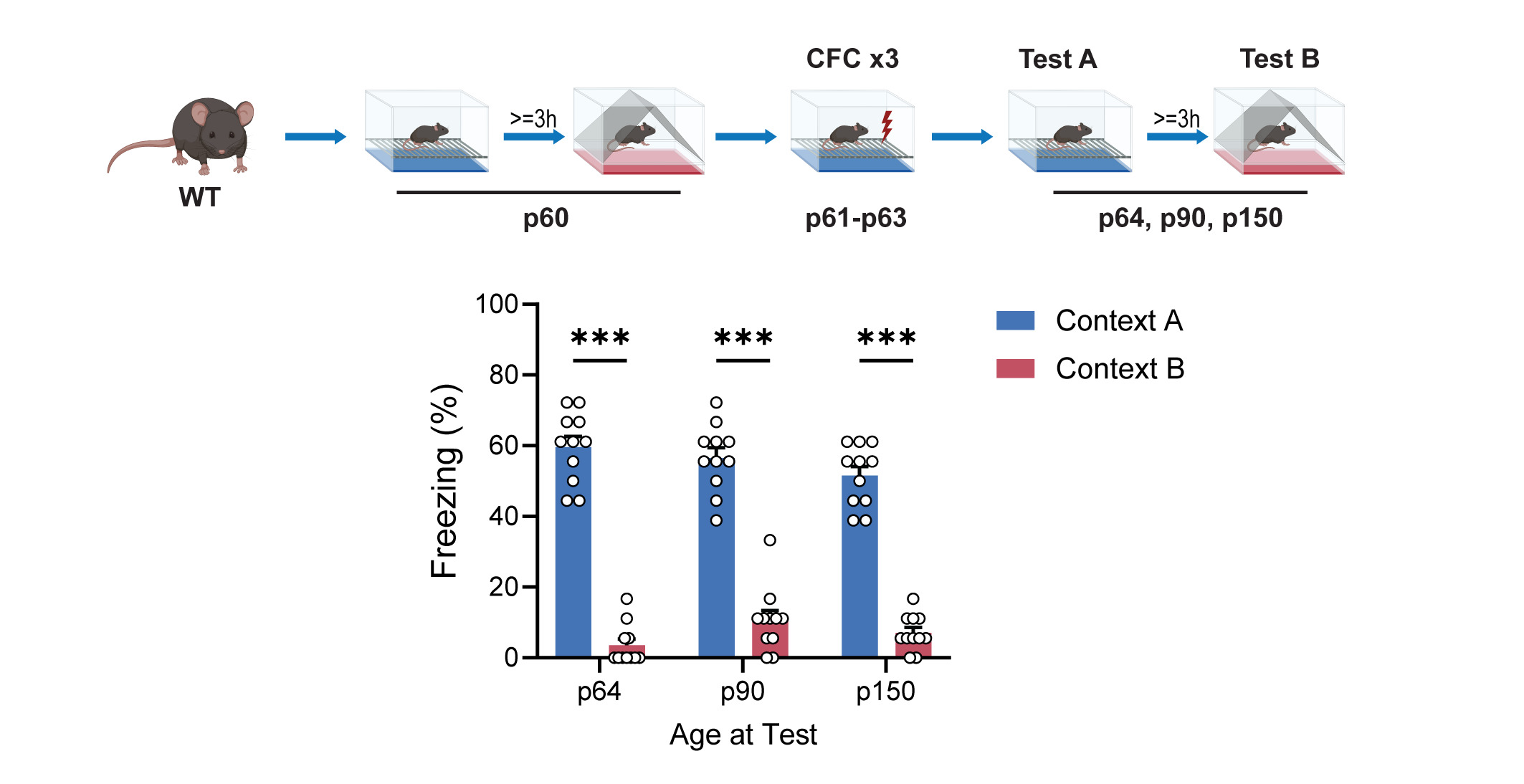

Supplement: S4 Fig — Contextual discrimination task performed on p60 mice shows a prolonged memory specificity in distinguishing dissimilar contexts (same setup used in this study for p28 mice, without tone) up to p150 (two-way RM ANOVA: Age of test: F(1.890, 18.90) = 2.209 p = 0.139, Context: F(1, 10) = 370.2 p < 0.001, Age of test × Context: F(1.897, 18.97) = 2.532 p = 0.108). Experimental diagrams were created in BioRender. Zhang, H. (2026) https://BioRender.com/qmq1etq. Data represent mean ± s.e.m., ***p < 0.001. The data underlying this Figure can be found in S1 Data. (TIF) [file pbio.3003908.s004.tif]

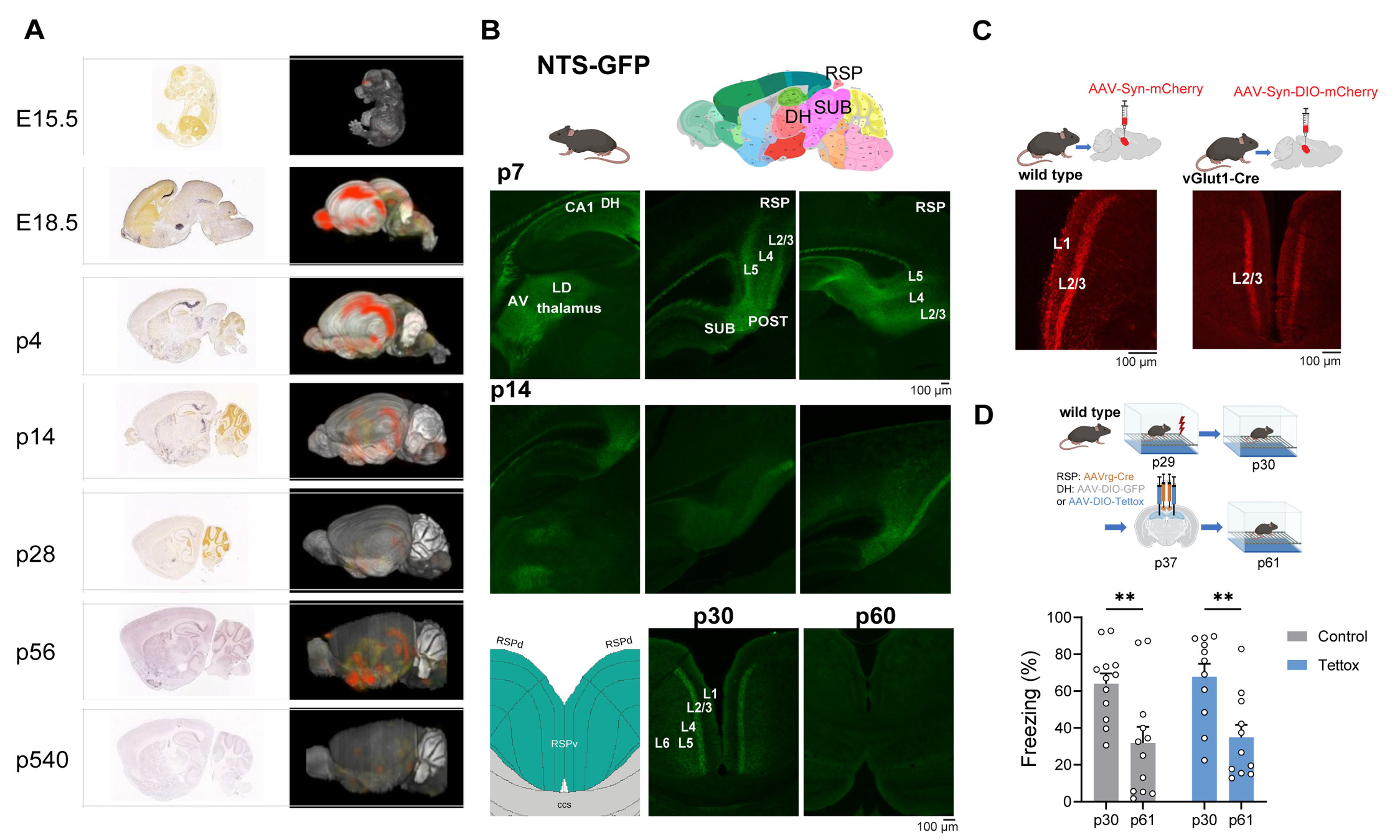

Supplement: S5 Fig — (A) Left, Delineation of brain areas showing strong NTS labeling in thalamic, hippocampal, and RSP regions during early development (p7–p14) in NTS-GFP mice [46]. Right, Representative images of NTS labeling showing two projections from SUB to RSP terminating in L2/3 and L5. (B) Disappearance of NTS from DH to RSP projections between p30 and p60. (C) Mature DH to RSP projections to RSP L1 and L2/3 (left), with excitatory projections terminating in L2/3 (middle) and lack of terminals in L5 (right). (D) Inhibition of DH to RSP projections does not affect the impaired retrieval of adolescent memories (n = 12, two-way RM ANOVA: Tettox: F(1,21) = 0.1627 p = 0.691, Age of test: F(1, 21) = 30.14 p < 0.001 Infusion × Age of test: F(1,21) = 0.0040 p = 0.950; Šídák’s post-hoc test: Control p30 vs. p61: p = 0.002, Tettox p30 vs. p61: p = 0.002). Experimental diagrams were created in BioRender. Zhang, H. (2026) https://BioRender.com/m7zoc9h. Data represent mean ± s.e.m., **p < 0.01. The data underlying this Figure can be found in S1 Data. (TIF) [file pbio.3003908.s005.tif]

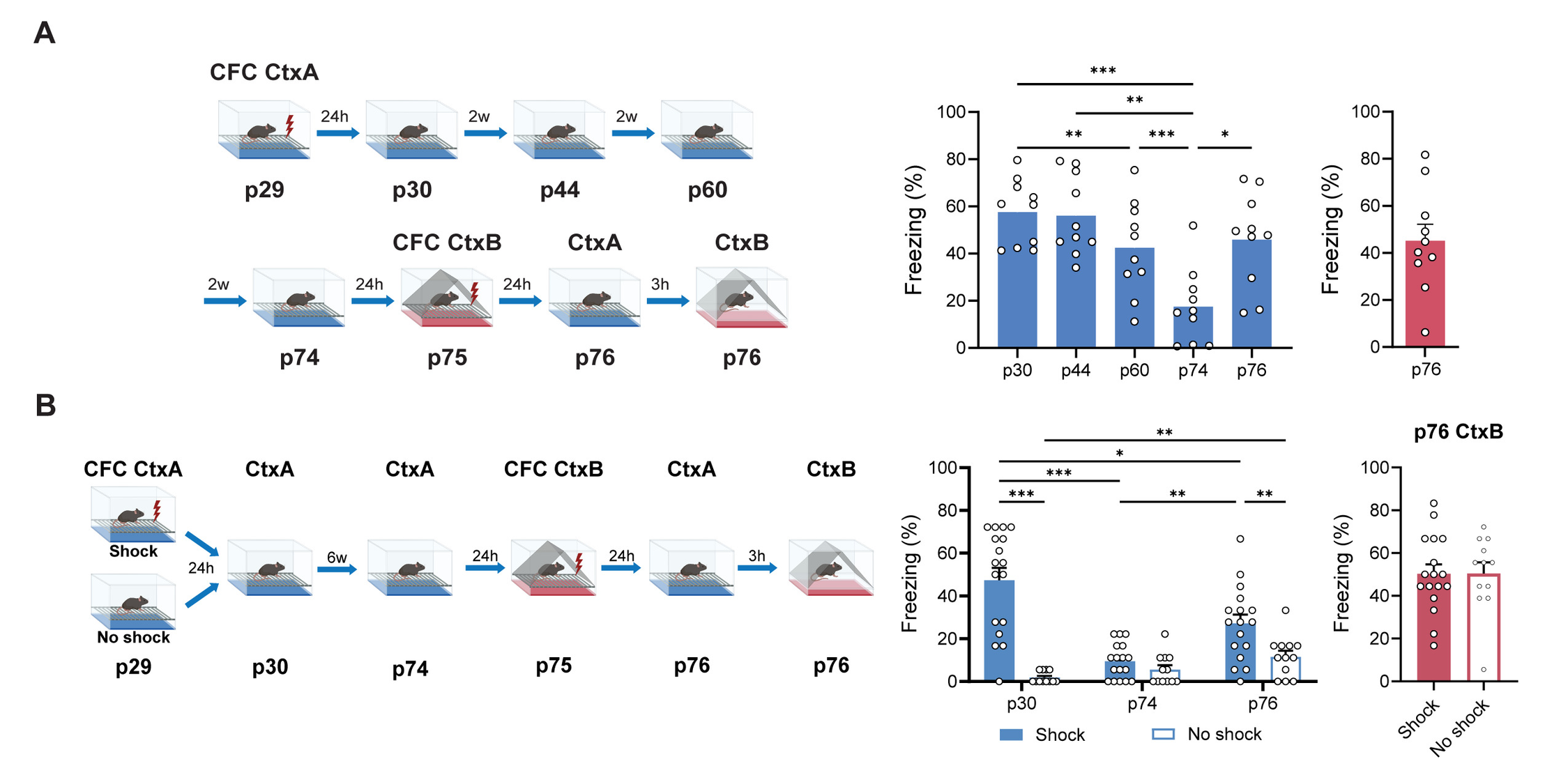

Supplement: S6 Fig — (A) Left, Experimental paradigm; Right, Shock in a novel context (Context B, CtxB) on p75 resulted in Context B-specific freezing, but also in increased freezing to the conditioned context (Context A, CtxA) (One-way RM ANOVA, F(2.431, 21.88) = 15.01, p < 0.001, Tukey’s post-hoc test: p30 vs. p60: p = 0.004, p30 vs. p74: p < 0.001, p44 vs. p74: p = 0.002, p60 vs. p74: p < 0.001, p74 vs. p76: p = 0.011). (B) The experiment was repeated with a no-shock control group, and no tests performed between p30 and p74. Shock in CtxB on p75 did not increase freezing in CtxA in no shock group(n = 17 for shock group, n = 12 for no shock group; two-way RM ANOVA: Test: F(1.657, 44.75) = 10.22, p < 0.001, Shock: F(1, 27) = 51.12, p < 0.001, Test × Shock: F(1.657, 44.75) = 15.35, p < 0.001; Tukey’s post-hoc test: shock group: p30 vs. p74: p < 0.001, p30 vs. p76: p = 0.041 p74 vs. p76: p = 0.004, no shock group: p30 vs. p76: p = 0.008, shock vs. no shock: p30: p < 0.001, p76: p = 0.005). Experimental diagrams were created in BioRender. Zhang, H. (2026) https://BioRender.com/wgbkg4j. Data represent mean ± s.e.m. ***p < 0.001; **p < 0.01; *p < 0.05. Mice that shocked on p30 and exhibited less than a 20% decline in freezing at p75 relative to p30 were excluded. See Fig 3C for data without this exclusion. The data underlying this Figure can be found in S1 Data. (TIF) [file pbio.3003908.s006.tif]

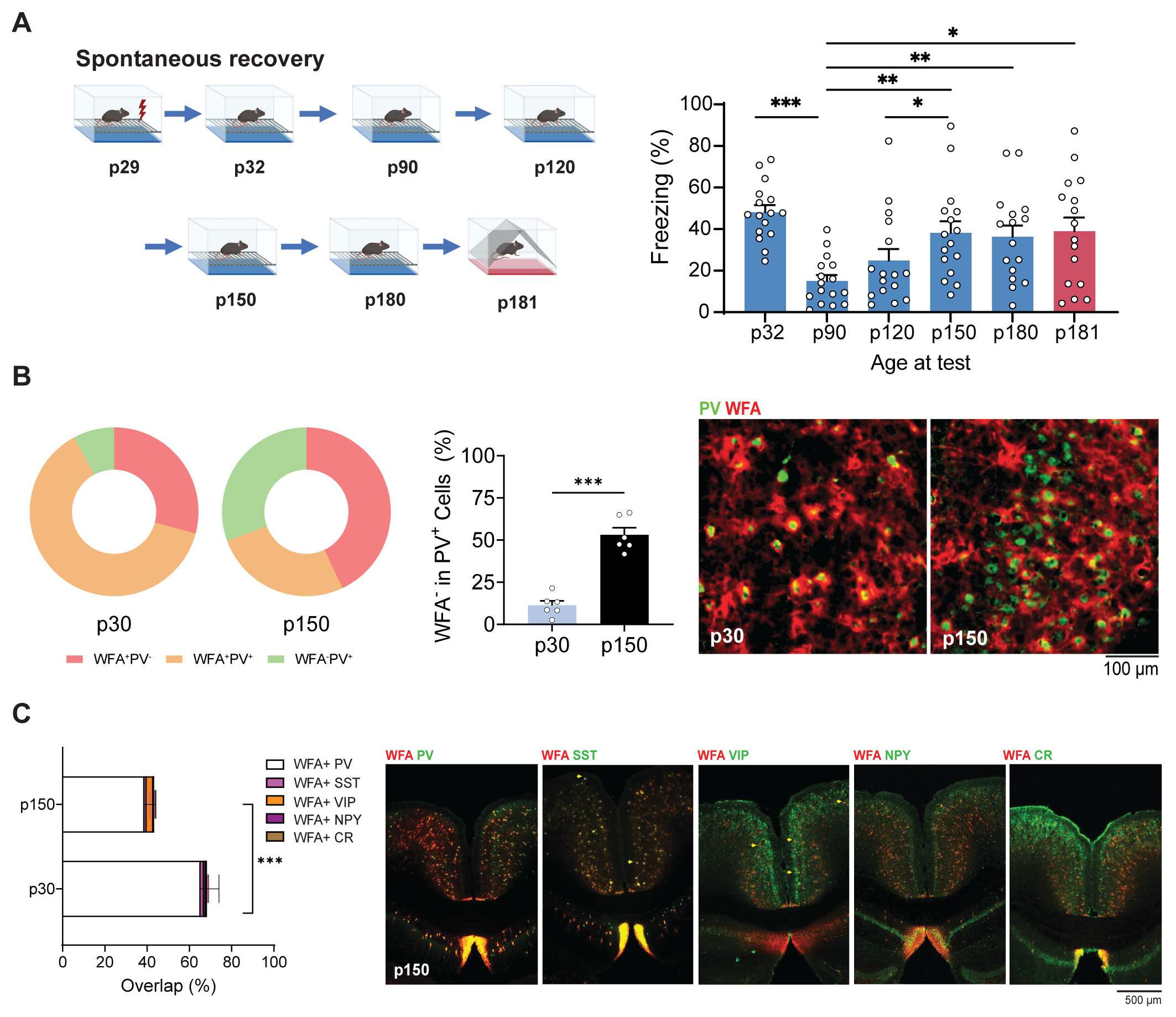

Supplement: S7 Fig — (A) Spontaneous recovery of adolescent aversive context memory by mid-adulthood/p150 (n = 16; one-way RM ANOVA: F = 8.025, p < 0.001; Tukey’s post-hoc test: p30 vs. p90: p < 0.001, p90 vs. p150: p = 0.002, p90 vs. p180: p = 0.012). (B) Increased proportion of RSP PNN-free PV interneuron in p150 compared to p30 (n = 6; unpaired two-tailed t test: t10 = 8.539, p < 0.001). (C) Decreased co-localization between PV and PNNs was not due to increased PNN formation around other interneuron classes, which showed a significant lack of co-localization with WFA relative to PV neurons at p30 and p150 (two-way ANOVA: Interneuron type: F(4, 50) = 560.8, p < 0.001 Age: F(1, 50) = 33.53, p < 0.001, Interaction: F(4, 50) = 39.44, p < 0.001, Šídák’s post-hoc test: p30 vs. p150: PV: p < 0.001, SST: p = 0.999, VIP: p = 0.690, NPY: p > 0.999, CR: p > 0.999). Experimental diagrams were created in BioRender. Zhang, H. (2026) https://BioRender.com/9yzhxu7. Data represent mean ± s.e.m. ***p < 0.001; **p < 0.01; *p < 0.05. The data underlying this Figure can be found in S1 Data. (TIF) [file pbio.3003908.s007.tif]

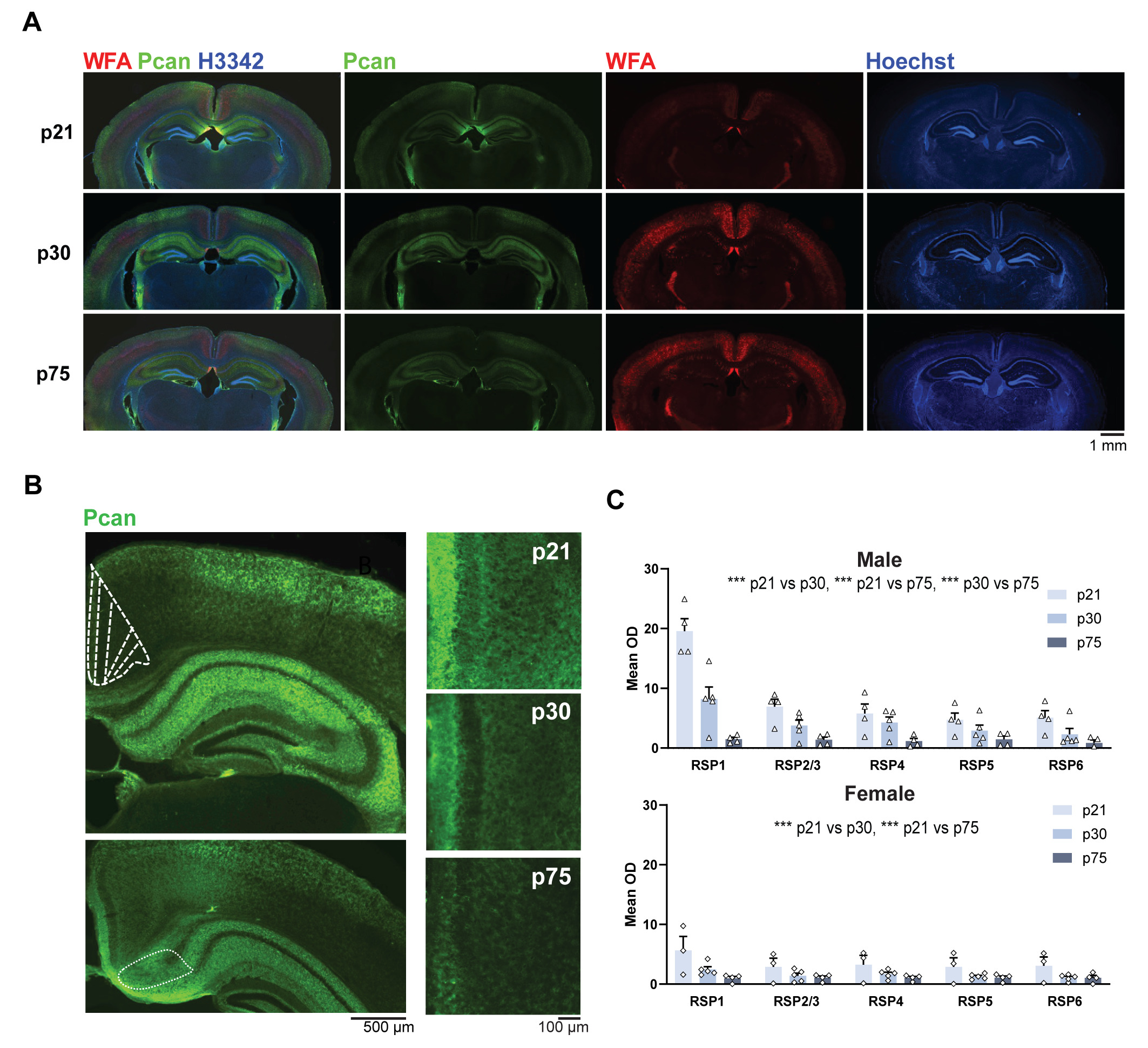

Supplement: S8 Fig — (A) Low magnification images demonstrating the cortical, hippocampal, and thalamic distribution of phosphacan and WFA at p21 (males n = 4, females n = 3), p30 (males, n = 5, females n = 5), and p75 (males n = 4, females n = 4). At p30, the highest levels are obtained in layer 1, containing the cortical axons. (B) High magnification images showing progressive post-adolescent down-regulation of phosphacan across layers and ECM compartments including but not restricted to PNNs. (C) Layer-by-layer quantification of phosphacan optical density demonstrating significant effects in males (two-way ANOVA: Age: F(2, 50) = 42.32, p < 0.001, Layer: F(4, 50) = 17.70, p < 0.001, Age × Layer: F(8, 50) = 6.574, p < 0.001, Tukey’s post-hoc test: p21 vs. p30: p < 0.001, p21 vs. p75: p < 0.001, p30 vs. p75: p < 0.001) and females (two-way ANOVA: Age: F(2,45) = 12.36, p < 0.001, Layer: F(4,45) = 1.478, p = 0.225, Age × Layer: F(8,45) = 0.5702, p = 0.797, Tukey’s post-hoc test: p21 vs. p30: p < 0.001, p21 vs. p75: p < 0.001). Data represent mean ± s.e.m., ***p < 0.001; **p < 0.01; *p < 0.05. The data underlying this Figure can be found in S1 Data. (TIF) [file pbio.3003908.s008.tif]

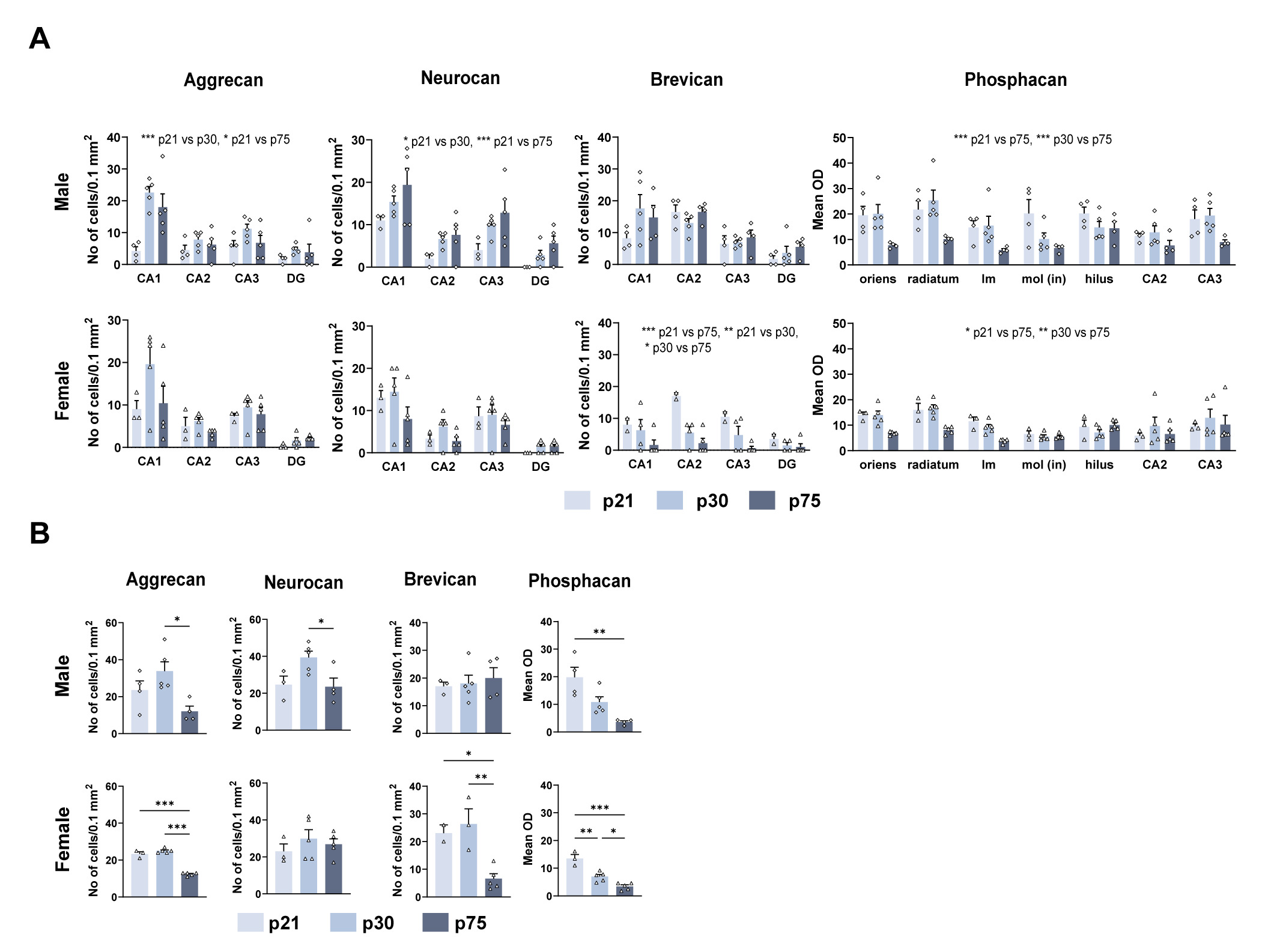

Supplement: S9 Fig — (A) Developmental decreases of PNN composition in DH (male aggrecan: p21: n = 4, other: n = 5, two-way ANOVA: Age: F(2, 44) = 12.14, p < 0.001, Area: F(3, 44) = 15.71, p < 0.001, Age × Area: F(6, 44) = 3.146, p = 0.012, Tukey’s post-hoc test: p21 vs. p30: p < 0.001, p21 vs. p75: p = 0.011; female aggrecan: p21: n = 3, other: n = 5, two-way ANOVA: Age: F(2, 40) = 3.515, p = 0.039, Area: F(3, 40) = 14.21, p < 0.001, Age × Area: F(6, 40) = 1.438, p = 0.225; male neurocan: p21: n = 3, other: n = 5, two-way ANOVA: Age: F(2, 40) = 9.519, p < 0.001, Area: F(3, 40) = 17.99, p < 0.001, Age × Area: F(6, 40) = 0.2076, p = 0.972, Tukey’s post-hoc test: p21 vs. p30: p = 0.027, p21 vs. p75: p < 0.001; female neurocan: p21: n = 3, other: n = 5, two-way ANOVA: Age: F(2, 40) = 2.812, p = 0.072, Area: F(3, 40) = 16.57, p < 0.001, Age × Area: F(6, 40) = 0.7006, p = 0.651; male brevican: p30: n = 5, other: n = 4, two-way ANOVA: Age: F(2, 40) = 1.567, p = 0.221, Area: F(3, 40) = 15.22, p < 0.001, Age × Area: F(6, 40) = 1.302, p = 0.279; female brevican: p21: n = 2, p30: n = 4, p75: n = 5, two-way ANOVA: Age: F(2, 32) = 15.37, p < 0.001, Area: F(3, 32) = 4.618, p = 0.009, Age × Area: F(6, 32) = 1.735, p = 0.145, Tukey’s post-hoc test: p21 vs. p30: p = 0.006, p21 vs. p75: p < 0.001, p30 vs. p75: p = 0.038; male phosphacan: p21: n = 4, other: n = 5, two-way ANOVA: Age: F(2, 70) = 20.37, p < 0.001, Area: F(6, 70) = 3.190, p = 0.008, Age × Area: F(12, 70) = 1.307, p = 0.235, Tukey’s post-hoc test: p21 vs. p75: p < 0.001, p30 vs. p75: p < 0.001; female phosphacan: p21: n = 3, other: n = 5, two-way ANOVA: Age: F(2, 70) = 7.449, p = 0.001, Area: F(6, 70) = 5.615, p < 0.001, Age × Area: F(12, 70) = 1.868, p = 0.054, Tukey’s post-hoc test: p21 vs. p75: p = 0.017, p30 vs. p75: p = 0.002). (B) Developmental change of PNN composition in SUB (male aggrecan: p30: n = 5, other: n = 4, one-way ANOVA: F = 5.746, p = 0.022; Tukey’s post-hoc test: p30 vs. p75: p = 0.017; female aggrecan: p21: n = 3, other: [file pbio.3003908.s009.tif]
